# Emotional cherry picking: the role of personality and goal orientation in selective emotion regulation for musical practice

**DOI:** 10.3389/fpsyg.2023.1201442

**Published:** 2023-07-27

**Authors:** Gerard Breaden Madden, Steffen A. Herff, Scott Beveridge, Hans-Christian Jabusch

**Affiliations:** ^1^Institute of Musicians’ Medicine (IMM), University of Music Carl Maria Von Weber, Dresden, Germany; ^2^The MARCS Institute for Brain, Behaviour and Development, Western Sydney University, Sydney, NSW, Australia

**Keywords:** emotion regulation, musical practice, personality, trait-dependent, mastery goal

## Abstract

**Introduction:**

Emotion regulation is an important part of optimising performance and successful goal pursuit in practice-based tasks such as making music. Musicians may regulate their own emotions during the course of their musical practice in order to improve their performance and ultimately attain their practice-related goals. The specific emotions they target may depend upon their personality traits but may also relate to the nature of their goal orientation, and the interaction between the two. This study investigates whether the emotions desired by musicians in their musical practice were dependent on their personality traits and Mastery goal orientation (the desire to master musical and technical skills).

**Methods:**

Via an online questionnaire, 421 musicians completed a personality scale and answered questions relating to their mastery practice goals. They also completed emotion scales indicating how strongly they desired to increase or decrease the intensity of specific emotions when practicing.

**Results:**

Overall, musicians preferred to up-regulate positive rather than negative emotions [*paired t*(420) = 58.13, *p* < 0.001]. Bayesian Mixed Effects models showed that personality traits affected musicians’ desire to regulate specific emotions. For example, higher levels of Agreeableness predicted greater desire to increase positive but not negative emotions, whereas Extraversion predicted greater desire to increase anger [*Est*. = 0.05, *SE* = 0.03, *Odds* (Est. > 0) = 43.03] but not positive emotions. The inclusion of Mastery goal orientation either amplified or mitigated these effects in several cases, and also introduced new trait-emotion relationships. Findings confirm a general hedonic principle underlying the emotions musicians desired in their musical practice. However, predicted by personality traits, musicians also sometimes sought to increase the intensity of unpleasant emotions.

**Discussion:**

These findings complement existing research that suggests that some Mastery-oriented musicians may seek an emotional state consisting of both positive and negative emotions. This and future studies on this topic may contribute to a better understanding of individual differences in emotion regulation ability as a potential aspect of individualised musical practice strategies.

## 1. Introduction

In many environments, it is desirable and sometimes even necessary to optimise our performance. Scenarios such as sport competitions and university exams demand performance at a high level in order to succeed and advance. As emotions have a strong influence on our thoughts and behaviour, the process of regulating our emotions may help us to optimise our performance (Baumeister et al., 2007; Hanin, 2010; Lane, 2012). Emotion regulation is thought to help us cope with situational demands (Kobylińska and Kusev, 2019) and this in turn may allow us to direct our goal-related behaviour—to pursue the targets and outcomes that are important for us (Aldao et al., 2015). In that sense, emotion regulation is a valuable psychological skill (Beedie et al., 2000), and it is important to understand the various factors that contribute to the regulation process.

### 1.1. The desired emotional state

In this research, we concentrate on an aspect of the emotion regulation process called the *Desired Emotional State (DES).* This is an individual’s preferred end-state related to their affect (Augustine et al., 2010), which is different from their *actual* affect—the emotions they experience in reality (Mannell et al., 2014). Emotion research often emphasises that individuals desire to maximise positive and minimise negative emotions (Larsen and Prizmic, 2004; Tamir et al., 2008). As emotions must be regulated in order to support psychological health and wellbeing (Aldao et al., 2010), a hedonic perspective governs much of the field of emotion regulation. Research indicates that individuals have stronger preference to experience pleasant over unpleasant affect (Rusting and Larsen, 1995; Feldman Barret, 1996; Diener, 2000; Augustine et al., 2010; Lane et al., 2011) and more often pursue pro-hedonic rather than contra-hedonic goals (English et al., 2017). However, as the world we live in is fast-paced and ever-changing, the goals we pursue can vary substantially in terms of their complexity, duration, required resources, and outcome. Goals may also vary both in time and between individuals. It follows then that there is also variation in the emotions individuals desire when they meet the challenges of different goals (Tsai et al., 2007; Tamir, 2011; Scheibe et al., 2013). This variety in desired emotions in turn motivates the deployment of many different kinds of emotion regulation strategies that may help an individual to cope with goal-specific demands (Kobylińska and Kusev, 2019). Emotion regulation strategies may be adaptive (e.g., reappraisal) or maladaptive (e.g., rumination) and are often aimed at the regulation of different and specific emotions. Accordingly, hedonic-oriented emotion regulation does not cover the entire spectrum of emotion regulation processes (Gross, 2015; Tamir et al., 2015; Ortner et al., 2018), and studies from contexts including sport, gaming, and music have shown that individuals may sometimes prioritise task-specific positive outcomes rather than experiencing positive emotions. In general, this is referred to as instrumental emotion regulation. An instrumental emotion regulation process may sometimes even involve selecting unpleasant emotions if those emotions are associated with a positive outcome (i.e., task-specific success). For example, Lane et al. (2011) showed that while most runners preferred to feel good while running, a minority of athletes believed that they would perform better in a competitive race if they used strategies aimed at increasing anger and even anxiety. In a computer game scenario, Tamir et al. (2008) found that individuals who pursued a confrontational goal were more inclined to experience anger in preparation of the task compared to individuals who completed a cooperative game goal. In the context of musical practice, Breaden Madden and Jabusch (2021) showed that some musicians selected a “mixed” emotional state (consisting of several strongly up-regulated pleasant emotions combined with moderate up-regulation of anger and nervousness) in order to support expertise-related practice goals. This same study also showed that some musicians held specific beliefs that certain unpleasant emotions may help to improve the quality of their music practice. In sum, these findings somewhat challenge the emphasis on individuals’ desire for positive, hedonic emotions. The implication of these findings is that, depending on an individual’s preferred outcome and whichever emotions they associate with the attainment of that outcome, a DES may consist of positive emotions, negative emotions, or even a mix of both.

### 1.2. The relationship between personality and DES

Emotion researchers often strive to understand emotion regulation behaviour as it occurs in different applied contexts. Musical practice is one such context that seems particularly relevant to study. Musical practice involves the processing and integration of multisensory information and the development of highly complex sensorimotor skills with an extreme spatiotemporal precision (for a review, see Münte et al., 2002). In this study, we examine the connections between individual differences and the emotions desired by musicians in their musical practice. Research frequently looks to individual characteristics to help explain variation in emotional experiences and emotion regulation behaviour (Eldesouky and English, 2018). Personality traits in particular are ideal to study in this regard, as they have strong links to emotion processing and affect regulatory abilities (Ivcevic and Brackett, 2014). Much of our understanding in this area concerns personality traits from the Five Factor Model of personality (Costa and McCrae, 1992). However, we have to consider two particular aspects of the literature in this field. The first aspect is that although studies that explore the relationship between personality traits and emotions are informative, they often concern *actual* rather than *desired* emotions. Whether the specific relationships between traits and desired emotions are the same as those between traits and actual emotions however, is unclear (Rusting and Larsen, 1995). Given the relative stability of the findings in this area, we can expect that personality traits may also contribute to the content of a desired emotional state as well as to an actual one. The second aspect of this literature we must consider is that available material is uneven. Most studies in this area tend to focus specifically on Extraversion and Neuroticism. This is perhaps due to these traits possessing a strong affective component (DeNeve and Cooper, 1998; Kotov et al., 2010; Brooks et al., 2020) in addition to their greater relevance to mental health and wellbeing when compared to other traits. In general, Extraversion is associated with greater tendency to experience pleasant and high-arousal affect (e.g., Costa and McCrae, 1989; Kampfe and Mitte, 2009) and is correlated positively with longer duration and stronger intensity of positive emotional episodes (Verduyn and Brans, 2012). Neuroticism, on the other hand, is typically associated with greater tendency to experience unpleasant affect (Watson and Clark, 1992; Rusting and Larsen, 1995) and is positively correlated with greater duration and intensity of negative emotional episodes (Verduyn and Brans, 2012).

With respect to other personality characteristics, individuals who are more conscientious (i.e., more disciplined and mindful) have better psychological health and experience fewer mental health and substance use disorders compared to less conscientious individuals (Malouff et al., 2005; Ruiz et al., 2008; Kotov et al., 2010; Fayard et al., 2012). These disorders are themselves associated with higher levels of negative emotions (Aldao et al., 2010). Higher Agreeableness is positively associated with greater levels of low-arousal, positive affect (Augustine et al., 2010) as well as greater use of emotion-improvement regulation strategies such as mindfulness and reappraisal (Baranczuk, 2019). Based on these studies, one could suppose that individuals high in either of these traits may desire greater levels of pleasant affect. Openness does not necessarily relate to a desire for any specific type of affect (Augustine et al., 2010). Although Openness has a clear affective component, McCrae (2007) suggests that this quality of Openness is largely reactive, reflecting an individual’s diverse range of emotions in response to their seeking of emotionally stimulating environments and activities.

### 1.3. The current study: trait- and goal-related DES

There is much information available concerning the emotional experiences of musicians. However, this information is often based on performance rather than practice contexts. As musical practice is not necessarily always an enjoyable experience (Lehmann and Ericsson, 1998), the first aim of this study was to investigate which emotions musicians *actually* experienced in their practice, and which emotions they *desired*. The second aim was to investigate whether musicians’ personality traits modulated/predicted their desire to regulate the intensity of their practice-related emotions. In addition to the predictive potential of a personality trait, the relationship between a trait and a desired emotion may also be considered in terms of its trait-*consistency/inconsistency.* This refers to the directionality of the relationship between the trait and the emotion. The relationship is theoretically trait-consistent if the regulated emotion matches the quality of a personality trait (Tamir, 2005). In the case of a highly extraverted individual for example, emotions that are positive and high-energy are trait-consistent. For an individual who is highly neurotic, in contrast, unpleasant emotions are trait-consistent. Trait-consistency is an important aspect of trait-related emotion regulation because it has implications for performance quality (Costa and McCrae, 1980; Gendolla, 2000; Ford and Tamir, 2014). For example, Tamir (2005) showed that when motivated to pursue a performance goal, more (vs. less) neurotic individuals selected trait-consistent unpleasant emotions due to the performance benefits they associated with these emotions. Furthermore, individuals who were more (vs. less) neurotic performed faster on decision-making tasks when in a trait-consistent negative (vs. neutral) emotional state. This was also the case for extraverts (vs. introverts) who also performed faster on the same decision-making tasks when in a trait-consistent positive (vs. neutral) state. Leung et al. (2014) showed that more (vs. less) neurotic individuals reported greater preference for recalling worrisome events in preparation for a challenging creativity task. When a worrisome rather than happy event was recalled, participants with higher Neuroticism scores generated more creative and flexible designs when given a cognitively demanding creativity task. The potential performance benefits of trait-consistent affect may arise because this state represents an achievable goal-related emotion state. It is achievable because it can be readily reached by one’s recurring life activities; daily-life studies suggest that individuals spend more time in situations that ‘match’ their prominent personality traits and self-concept (Diener, 1984). Furthermore, individuals may be motivated to use goal-pursuit mechanisms that are congruent with their stable motivational orientation (Higgins, 2000). In theory then, trait-consistent affect might be especially preferable in contexts that are particularly challenging or require performance at a high level (Gendolla, 2000). In musical contexts, many musicians pursue the goal of Mastery (acquiring expertise in musical knowledge and instrumental or vocal skills). Mastery is a challenging target, requiring years of sustained, deliberate practice (Ericsson et al., 1993) and the development and honing of highly complex motor programs and problem-solving strategies. With this in mind, the final aim of the current research was to investigate whether Mastery goal orientation interacted with personality traits to further shape the content of a musical practice-related DES.

In light of existing research and with respect to the outlined aims of this research, we propose the following hypotheses: *H*_1_: Musicians will have stronger desire for pleasant over unpleasant emotions in their musical practice. *H*_2_: Musicians’ personality traits will modulate/predict their desire to regulate the intensity of practice-related emotions*:* As existing literature is uneven, it is not possible to make equally precise predictions concerning the relationship between desired emotions and each individual trait. Thus, we opted to interpret findings for each trait according to different evaluative criteria based on prior literature support (please see the *Statistical Approach* section). *H*_3_: Mastery goal orientation will modulate trait-dependent effects to further shape the content of a musical practice-related DES (*Mastery-Related DES).*

## 2. Materials and methods

### 2.1. Participants

This study used data collected but only partly analyzed from an earlier study on emotion regulation behaviour in musical practice (Breaden Madden and Jabusch, 2021). A total of 421 musicians completed an online questionnaire distributed via their host music institution (conservatoire, orchestra, university of music, etc.). Participants were recruited from the USA (113), UK (72), Germany (64), Norway (42), Austria (26), Denmark (14), Ireland (14), and thirteen other countries (≤10, respectively).

### 2.2. Materials

Participants completed an online modular questionnaire in English. This questionnaire is described in detail in Breaden Madden and Jabusch (2021). Only those modules of the questionnaire relevant to the present study are described below.

#### 2.2.1. Demographics and musical expertise

Participants reported various aspects of their musical expertise and training. Items include the age at which they began playing music (Age of Commencement; AoC), total number of years playing music (Years of Playing: YoP), number of days per week spent playing music (DpW), Cumulative Life Practice time (CLP; derived from retrospective self-reported year-by-year practice hours), status as a student or professional musician, and the styles of music in which they are actively involved. These measures successfully capture aspects of musical expertise that carry predictive value for music-related tasks (Ericsson et al., 1993; Davidson et al., 1996; Hallam, 1998; Beveridge et al., 2020; Buck et al., 2021).

#### 2.2.2. Personality

Participants completed the Ten Item Personality Inventory (TIPI; Gosling et al., 2003) The TIPI is a personality measure based on the Five Factor Model (Costa and McCrae, 1985) which assesses five personality dimensions: Agreeableness, Conscientiousness, Emotional Stability (a trait analogue to inverse Neuroticism), Extraversion, and Openness. The TIPI has demonstrated adequate factor structure, test-retest reliability, and both convergent and discriminant validity with established personality measures such as the NEO-PI-R (Costa and McCrae, 1992; Muck et al., 2007; Renau et al., 2013; Chiorri et al., 2015). The Cronbach’s α-coefficient for each scale was 0.78 (Agreeableness), 0.71 (Conscientiousness), 0.86 (Emotional Stability), 0.74 (Extraversion), and 0.76 (Openness), indicating good internal consistency for all scales (Nunnally, 1978).

#### 2.2.3. Actual emotions and Desired Emotional State (DES)

Participants completed three emotion scales. In the first scale they reported the emotions they actually experienced in musical practice (“*How strongly do you typically experience each of the following emotions during musical practice*”). The second and third scales concerned how much they desired to either increase or decrease the intensity of those same emotions in practice (“*How much would you like to increase the intensity of each emotion in order to best support your practice?*” and *“How much would you like to decrease the intensity of each emotion in order to best support your practice?”*. Responses were captured via seven point Likert scales (1 = not at all, 7 = a great deal). The three scales described above were based on the UWIST Mood Adjective Checklist (UMACL; Matthews et al., 1990) and included emotion terms derived from the circumplex model of emotion (Russell, 1980). The emotions assessed high- and low-arousal pleasant emotions (*Happiness, Calmness*), high-arousal unpleasant emotions (*Anger, Anxiety*), low-arousal unpleasant emotions (*Gloom, Downheartedness*), and emotions that vary in terms of energetic arousal (*Energy, Nervousness, and Sluggishness*). One emotion-adjacent term (*Focus/Concentration*) was also included. Previous research has investigated focus/concentration as an additional aspect of musicians’ practice-related mindsets (Hallam, 1995).

#### 2.2.4. Mastery goal orientation

Musicians’ desire to master musical and technical skills was captured via questionnaire items related to musical practice activities/attitudes and established via Principle Component Analysis (PCA). This factor was named “*Mastery*”. Higher factor scores represent stronger Mastery orientation and lower scores represent weaker Mastery orientation. Example items loading onto this factor included “*I practice difficult techniques or pieces until I have mastered them*” and “*It is very important to me to continue to perfect my musical and technical abilities*.” Participants rated each item on a seven point Likert scale according to how accurately each statement represented their attitude (1 = very untrue of me, 7 = very true of me). For a complete explanation of this factor and the associated procedure, please see Breaden Madden and Jabusch (2021).

### 2.3. Statistical approach

This analysis contained several steps. The first step involved examining participants’ musical expertise and practice-related emotions via descriptive statistics (median and frequency) and inferential statistics (paired *t*-tests). With respect to these emotions, we investigated the typical intensity at which musicians *actually* experienced emotions in their musical practice and how strongly they *desired* to increase or decrease the intensity of these same emotions. For the second step, we deployed two Bayesian Mixed Effects models to investigate whether personality traits predicted musicians’ desire to increase or decrease practice-related emotions, respectively. We used two models because the desires to increase and decrease the intensity of emotions do not necessary lie on the same continuum, and they were assessed using two different scales within the questionnaire. Hereafter, we refer to findings from this step in the analysis as *Trait-Dependent DES* effects. In step three, we deployed two additional Bayesian models to investigate whether Mastery goal orientation affected these trait-dependent DES effects. As before, the first of these additional models focussed on the desire to increase emotion intensity, and the second focussed on the desire to decrease emotion intensity. We refer to findings from this step of the analysis as *Mastery-Related DES* effects.

All models were implemented in R Core Team (2022) using the *brms* package (Bürkner, 2017). We fitted each model with a fixed effect for each predicted emotion as well as with the interaction between a specific emotion and each of the predictors. All models contained a random effect for participant and a weakly informative prior (a t-distribution with a mean of 0, a standard deviation of 1, and 3 degrees of freedom; see Gelman et al., 2008). All continuous predictors were scaled to have a mean of 0 and a standard deviation of 1. To investigate main effects and interactions, we report a combination of *directed* and *exploratory* hypothesis tests. These tests evaluate the evidence of a given effect to be smaller or larger than zero (Evidence Ratio), as well as the coefficient estimates (Estimate) and the error within this estimate (Error). In the case of *directed* hypothesis tests, we consider evidence ratios ≥19 analogous to significant evidence in favour of an effect under an alpha level of 0.05. For *exploratory* hypothesis tests, we consider evidence ratios ≥39 analogous to significant evidence for an effect. Evidence ratios of ≥19 and ≥39 are referred to as “strong” evidence (Milne and Herff, 2020).

We opted to report a combination of directed and exploratory hypothesis tests in light of the uneven quantity of research available for each specific personality trait. We used directed hypothesis tests for Extraversion and Emotional Stability (i.e., tests with a less conservative evidence threshold of ≥19; the Bayesian equivalent to a one-sided frequentist hypothesis test; Makowski et al., 2019; Marsman and Wagenmakers, 2017). We used exploratory hypothesis tests for the traits Agreeableness, Conscientiousness, and Openness (i.e., tests with a more conservative evidence threshold of ≥39; the Bayesian equivalent of a two-sided frequentist hypothesis test, Makowski et al., 2019; Marsman and Wagenmakers, 2017). The chosen prior, scaling, and evidence reference are commonly used in the music cognition and perception literature (Dobrowohl et al., 2019; Herff et al., 2020a,2021b; Jääskeläinen et al., 2020, MacRitchie et al., 2020; Smit et al., 2020; Cecchetti et al., 2021; Herff et al., 2021a,b; Smit and Milne, 2021). All scripts used in this analysis are available in Supplementary Appendices B, C.^1^

We used Bayesian statistics as this allows us to include prior uncertainty into our models (Van de Schoot and Depaoli, 2014). Importantly, this allows us to calculate and report the whole posterior probability distribution of each effect size and hypothesis, instead of only point-estimates of the most likely effect size – as a frequentist approach would do (Smit et al., 2019). In turn, we can then report the continuous evidence ratio observed for each effect and specify how we interpret such evidence, whilst allowing readers to evaluate the evidence to their preference. This differs greatly from reporting *p*-values that only allow binary but not continuous evidence weighting and interpretation (Wagenmakers et al., 2018). Lastly, this approach allows us to report credibility intervals. A 95% credibility interval indicates that, in light of the observed evidence, there is a 95% probability that the true (unknown) estimate would be within the calculated interval. We consider this more informative than a 95% confidence interval used in Null Hypothesis Statistical tests (NHST), which is interpreted as the interval that will capture the true parameter value in approximately 95% of repeated samples (Morey et al., 2016).

*Note: exploratory and directed hypothesis tests are statistical tests conducted as part of each overall Bayesian Mixed Effects model. As such, they do not correspond in a direct numerical manner to the numbered hypotheses as outlined in the introduction. Exploratory and directed tests can be seen as a collection of sub-hypotheses which contribute as a whole to the testing of H_2_ and H_3_.*

## 3. Results

### 3.1. Overview of the sample

The majority of musicians in this sample identified as female and as student musicians as opposed to professional musicians. The median age was 23 years. When they began to make music, their median age was 7 years. Since then they had a median of 16 years of playing experience. Detailed information on demographic and musical expertise data is provided in Table 1. Musicians who reported playing a bowed string, keyboard, or woodwind instrument were represented most strongly in this sample. This sample of musicians reported being most active in Classical and Post-1950’s Contemporary Classical music (involvement with different music styles was assessed via a seven point Likert scale where 1 = extremely inactive and 7 = extremely active).

**TABLE 1:** Musicians’ demographic and musical expertise data.

Total sample *N* = 421	Median (IQR) or *N*
Sex (F/M/O)	254/167/0
Affiliation (P/S)	120/301
Age (years)	23 (20, 26)
AoC (years)	7 (5, 9)
YoP (years)	16 (13, 20)
DpW	6 (5, 7)
CLP (1,000 h)	8.6 (4.3, 14.5)
**Musical Styles *(1* = *extremely inactive, 7* = *extremely active)***	
Post-1950s contemporary classical	5 (3, 6)
Classical	7 (5, 7)
Jazz	2 (1, 4)
Rock	2 (1, 2)
Pop	2 (1, 4)
**Primary musical instrument**	
Bowed String	105 (24.9%)
Keyboard	81 (19.2%)
Woodwind	67 (15.9%)
Voice	62 (14.7%)
Brass	51 (12.1%)
Plucked String	37 (8.7%)
Percussion	18 (4.2%)

Sex: F, female; M, male; O, other; Affiliation: P, professional; S, student; AoC, Age of Commencement; YoP, Years of Playing; DpW, number of days per week currently practicing music; CLP, Cumulative Life Practice time.

Table 2 provides an overview of musicians’ actual and desired emotions in musical practice. Musicians reported actually experiencing moderate-strong levels of pleasant emotions and low-moderate levels of unpleasant emotions. Overall, pleasant emotions were experienced to a significantly stronger level than unpleasant emotions [*paired t*(420) = 28.22, *p* < 0.001; 95% CI (−1.74, 0.15)].

**TABLE 2:** Overview of musicians’ *actual* and *desired* emotions in musical practice.

	Median (IQR)		
	Actual	Increase	Decrease
Anger	3 (1, 4)	1 (1,2)	4 (1, 7)
Anxiety	3 (2, 4)	1 (1,1)	6 (4, 7)
Calmness	5 (4, 6)	6 (4, 7)	1 (1,1)
Downheartedness	2 (1, 4)	1 (1,1)	7 (4, 7)
Energy	5 (4, 6)	6 (5, 7)	1 (1,1)
Focus/Concentration	6 (5, 6)	7 (6, 7)	1 (1,1)
Gloom	3 (2, 4)	1 (1,1)	6 (2, 7)
Guilt	2 (1, 4)	1 (1,1)	7 (4, 7)
Happiness	5 (4, 6)	5 (4, 7)	1 (1,1)
Nervousness	2 (1, 4)	1 (1,1)	6 (3, 7)
Sluggishness	3 (2, 4)	1 (1,1)	7 (4, 7)

Emotions were measured on a seven point Likert scale (1, not at all; 7 = a great deal). Actual, how strongly musicians typically experienced an emotion during musical practice. Increase, how strongly a musician desired to increase the intensity of an emotion during their musical practice. Decrease, How strongly a musician desired to decrease the intensity of an emotion during their musical practice. Minimum and maximum recorded response for all items: minimum = 1, maximum = 7.

In general, musicians indicated a strong desire to increase the intensity of pleasant emotions coupled with a strong desire to decrease the intensity of unpleasant emotions. Their observed desire to increase the intensity of pleasant emotions was significantly stronger than their desire to increase unpleasant emotions [*paired t*(420) = 58.13, *p* < 0.001; 95%; CI (−13.36, −12.07)]. Accordingly, their desire to decrease the intensity of unpleasant emotions was significantly stronger than the desire to decrease pleasant emotions [*paired t*(420) = −36.87, *p* < 0.001; 95% CI (28.87, 31.51)].

### 3.2. Bayesian Mixed Effects models: *Trait-Dependent DES—*the impact of personality traits on musicians’ desire to increase or decrease the intensity of practice-related emotions

Table 3 summarises the findings from the first two Bayesian models concerning musicians’ desire to increase and decrease emotions in musical practice. We named these models “*Emotion Increase”* and “*Emotion Decrease*.” These tables contain only the hypothesis tests with odds ratios ≥19 (marked *) for directed hypothesis tests, and ≥39 (marked ^**^) for exploratory tests, both referring to strong evidence for their respective hypotheses (Milne and Herff, 2020). The complete fitted model and all tested hypotheses are given in Supplementary Appendix A (for these and all subsequent Bayesian models). Positive coefficient estimates represent *greater* desire to increase or decrease an emotion, whereas negative estimates represent *less* desire to increase or decrease an emotion.

**TABLE 3:** Summary of directed and exploratory hypothesis tests for the effects of the *Emotion Increase* and *Emotion Decrease* models.

		Personality traits									
		Agreeableness		Conscientiousness		Emotional Stability		Extraversion		Openness	
		Increase	Decrease	Increase	Decrease	Increase	Decrease	Increase	Decrease	Increase	Decrease
Anger	*Estimate*	–	–	–	–	–	–	0.05	–	0.09	−0.2
	*Odds*	–	–	–	–	–	–	43.03*	–	>9999**	>9999**
Anxiety	*Estimate*	–	–	–	–	–	−0.01	–	–	–	–
	*Odds*	–	–	–	–	–	36.18*	–	–	–	–
Calmness	*Estimate*	0.1	–	–	–	−0.17	–	–	–	–	–
	*Odds*	240.38**	–	–	–	>9999*	–	–	–	–	–
Downheartedness	*Estimate*	–	–	–	–	–	–	–	–	–	–
	*Odds*	–	–	–	–	–	–	–	–	–	–
Energy	*Estimate*	0.13	–	–	–	−0.15	–	–	–	−0.12	–
	*Odds*	>9999**	–	–	–	>9999*	–	–	–	>9999**	–
Focus / Concentration	*Estimate*	0.08	–	–	–	–	–	0.06	–	–	–
	*Odds*	62.64**	–	–	–	–	–	54.34*	–	–	–
Gloom	*Estimate*	−0.09	–	–	–	–	−0.15	–	–	–	−0.1
	*Odds*	98.29**	–	–	–	–	594.74*	–	–	–	303.35**
Guilt	*Estimate*	–	–	–	–	–	–	–	–	–	–
	*Odds*	–	–	–	–	–	–	–	–	–	
Happiness	*Estimate*	0.13	–	–	–	−0.18	–	–	–	−0.15	–
	*Odds*	>9999**	–	–	–	>9999*	–	–	–	>9999**	–
Nervousness	*Estimate*	–	–	–	–	–	–	–	–	–	−0.12
	*Odds*	–	–	–	–	–	–	–	–	–	1165.67**
Sluggishness	*Estimate*	–	–	–	–	–	–	–	–	–	–
	*Odds*	–	–	–	–	–	–	–	–	–	–

*Indicates an Odds Ratio ≥ 19, which we deem strong evidence for an effect for all directed hypothesis tests. **Indicates an Odds Ratio ≥ 39, which we deem strong evidence for an effect for all exploratory hypothesis tests. Positive Estimate coefficient values represent stronger desire to increase or decrease an emotion. Negative Estimate coefficient values represent less strong desire to increase or decrease an emotion. All model estimates are standardised to SDs. This means that, for example, the Emotion Increase model predicts a reduction of 0.18 (Est. = −0.18) in the desire to increase Happiness for each 1 SD of Emotional Stability above the mean. The standard error of each estimated mean in this model can be found in Supplementary Appendix A1. The rows represent: the estimated mean of the standardized effect (*Estimate*); the evidence ratio in favour of the hypothesis [*Odds* (> or<0)].

#### 3.2.1. Emotion Increase

Higher Agreeableness predicted stronger desire to increase the intensity of calmness, energy, focus/concentration and happiness (positive estimate coefficients for each emotion). Higher Agreeableness also predicted less strong desire to increase the intensity of gloom (negative estimate coefficient). Higher Emotional Stability predicted less strong desire to increase calmness, energy, and happiness. Higher Extraversion predicted stronger desire to increase anger and focus/concentration. Higher Openness predicted stronger desire to increase anger, in addition to predicting less strong desire to increase focus/concentration, and happiness. The model provided no evidence that higher Conscientiousness predicted the desire to increase any of the assessed emotions.

#### 3.2.2. Emotion decrease

This model showed little to no evidence that Agreeableness, Conscientiousness or Extraversion predicted musicians’ desire to decrease the intensity of any of the assessed emotions. Higher Emotional Stability predicted less strong desire to decrease anxiety and gloom. Higher Openness predicted less strong desire to decrease anger, gloom, and nervousness.

**FIGURE 1:**
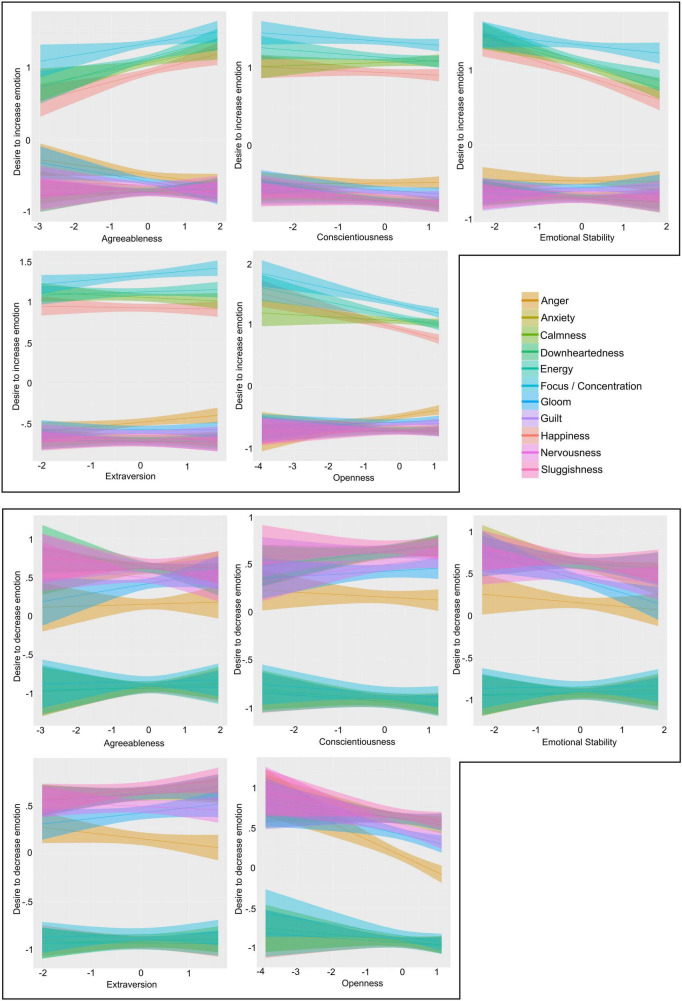
Marginal effects plots showing musicians’ predicted desire to regulate the intensity of practice-related emotions according to different levels of a personality trait. The five plots in the **upper panel** refer to musicians’ desire to *increase* the intensity of emotions. The five plots in the **lower panel** refer to musicians desire to *decrease* the intensity of emotions. Line colour indicates each specific emotion. Bands represent 95% Credible Intervals. All values normalised to Mean = 0; Standard Deviation = 1.

Figure 1 shows the marginal effects plots for both models. The upper panel refers to the *Emotion Increase* model, and the bottom panel refers to the *Emotion Decrease* model. An upward slope trajectory corresponds to a positive estimate coefficient, and a downward slope corresponds to a negative estimate coefficient. All values on the axes are normalized, and negative y-axis values represent less desire to increase (upper panel) or decrease (lower panel) an emotion, respectively. Whilst Table 3 provided an overview of effects for which we identified strong evidence (i.e., Odds Ratio ≥ 19 for directed hypotheses, and ≥ 39 for exploratory hypotheses), these plots show all of the assessed emotions in order to highlight their relative relationship to higher levels of each personality trait.

### 3.3. Bayesian Mixed Effects models: *Mastery-Related DES—*the interaction between Mastery goal orientation and personality traits on musicians’ desire to increase or decrease the intensity of practice-related emotions

To investigate whether Mastery goal orientation impacted trait-dependent effects on desired emotions, two additional models were deployed. In these models, the desire to either increase or decrease the intensity of each practice-related emotion was predicted by a Mastery*Trait interaction (i.e., one interaction term per individual trait). We named these models *Mastery Increase* and *Mastery Decrease.* These effects are shown in Table 4. As before, we report only hypothesis tests with an Odds Ratio ≥19 for directed hypothesis tests, and ≥39 for exploratory hypothesis tests. The estimates in Table 4 represent the magnitude of change of the effects seen in the initial *Emotion Increase* and *Emotion Decrease* models, depending on Mastery orientation.

**TABLE 4:** Summary of directed and exploratory hypothesis tests for the effects of the *Mastery Increase* and *Mastery Decrease* models.

		Personality trait × goal interaction									
		Agreeableness × Mastery		Conscientiousness × Mastery		Emotional stability × Mastery		Extraversion × Mastery		Openness × Mastery	
		Increase	Decrease	Increase	Decrease	Increase	Decrease	Increase	Decrease	Increase	Decrease
Anger	*Estimate*	–	–	–	–	–	–	–	−0.11	–	–
	*Odds*	–	–	–	–	–	–	–	399*	–	–
Anxiety	*Estimate*	–	–	–	–	–	–	–	–	–	–
	*Odds*	–	–	–	–	–	–	–	–	–	–
Calmness	*Estimate*	–	–	–	–	–	–	–	–	−0.05	–
	*Odds*	–	–	–	–	–	–	–	–	63.22**	–
Downheartedness	*Estimate*	–	–	–	–	–	–	–	–	–	0.08
	*Odds*	–	–	–	–	–	–	–	–	–	65.04**
Energy	*Estimate*	–	–	−0.06	–	–	–	–	–	–	–
	*Odds*	–	–	71.35**	–	–	–	–	–	–	–
Focus / Concentration	*Estimate*	0.08		−0.06	–	–	–	−0.07	–	–	–
	*Odds*	61.08**	–	110.55**	–	–	–	284.71*	–	–	–
Gloom	*Estimate*	0.09	–	–	–	−0.12	0.14	–	−0.07	−0.06	0.12
	*Odds*	115.67**	–	–	–	1999*	332.33*	–	23.93*	98.64**	>9999**
Guilt	*Estimate*	–	–	–	–	–	–	–	–	–	0.08
	*Odds*	–	–	–	–	–	–	–	–	–	77.65**
Happiness	*Estimate*	–	–	−0.09	–	−0.08	–	−0.1	–	–	–
	*Odds*	–	–	1856.67**	–	94.24*	–	>9999*	–	–	–
Nervousness	*Estimate*	–	–	–	–	–	–	–	–	–	–
	*Odds*	–	–	–	–	–	–	–	–	–	–
Sluggishness	*Estimate*	–	–	–	–	–	–	–	–	–	0.09
	*Odds*	–	–	–	–	–	–	–	–	–	160.85**

*Indicates an Odds Ratio ≥ 19. **Indicates an Odds Ratio ≥ 39. All model estimates are standardised to SDs. This means that, for example, the “Emotion Increase” model (see Table 3) predicts a reduction of 0.18 (Est = −0.18) in the desire to increase Happiness for each 1 SD of Emotional Stability above the mean. According to the Mastery Increase model, if a hypothetical musician showed a Mastery orientation 1 SD above the mean, then this model would predict an additional 0.08 (Est = −0.08) reduction in the desire to increase Happiness (for a total of −0.24) per SD Emotional Stability above the mean. The standard error of each estimated mean in this model can be found in Supplementary Appendix A2. The rows represent: the estimated mean of the standardized effect (*Estimate*); the evidence ratio in favour of the hypothesis [*Odds* (> or <0)].

#### 3.3.1. Mastery and DES increase

As Mastery orientation increased, higher Agreeableness predicted increasingly greater desire for focus/concentration and gloom. As mastery orientation increased, higher Conscientiousness predicted reduced desire to increase energy, focus/concentration, and happiness. With increasing Mastery orientation, higher Emotional Stability predicted reduced desire to increase gloom, and happiness. As Mastery orientation increased, higher Extraversion predicted less desire to increase focus/concentration and happiness. Finally, as Mastery orientation increased, higher Openness predicted less desire to increase calmness and gloom.

#### 3.3.2. Mastery and DES decrease

With increasing Mastery orientation, higher Emotional Stability predicted greater desire to decrease the intensity of gloom. With increasing Mastery orientation, higher Extraversion predicted less desire to decrease anger and gloom. With increasing Mastery orientation, higher Openness predicted stronger desire to decrease downheartedness, gloom, guilt, and sluggishness. The model provided little to no evidence that with increasing Mastery orientation, Agreeableness and Conscientiousness predicted a change of musicians’ desire to decrease any of the assessed emotions.

## 4. Discussion

In this study, we investigated which emotions musicians desired to increase or decrease in their musical practice, whether their personality traits predicted their desire to regulate the intensity of these emotions, and how Mastery goal orientation shaped the predictive value of personality traits for these emotions. With respect to the hypotheses outlined in the introduction, we found the following:

(*H*_1_) In general, musicians desired pleasant emotions and did not desire unpleasant emotions in their musical practice. (*H*_2_) Bayesian Mixed Effects models indicated that personality traits predicted musicians’ desire to increase and/or decrease the intensity of both pleasant and unpleasant emotions. (*H*_3_) Mastery goal orientation modulated personality trait-dependent effects.

### 4.1. Emotions *actually* experienced, and those *desired*

Overall, the results of this study represent new findings concerning musical practice-related emotion states. In general, musicians’ actual emotions in musical practice emphasised pleasant over unpleasant emotions. This is consistent with studies which show that our daily lives are characterised in general more strongly by positive rather than negative emotions (Kahneman et al., 2004). Musicians’ DES also strongly emphasised pleasant over unpleasant emotions. This is also in keeping with the wider emotion regulation literature - it is rare for an individual to want to feel worse rather than better (Tsai et al., 2007), and evidence from sport and gaming contexts (Tamir et al., 2008; Lane et al., 2011) show that individuals are generally motivated to feel positive rather than negative emotions. In sum, although a musician’s *actual* emotions in musical practice may comprise a combination of pleasant and unpleasant emotions, their *desired* emotions tend to be positive. In other words, while musicians may not necessarily experience only positive emotions in their practice, they generally want to.

### 4.2. Trait-Dependent DES

#### 4.2.1. Agreeableness

Higher Agreeableness predicted stronger desire to increase pleasant emotions coupled with less desire to increase unpleasant emotions. Individuals who score highly on Agreeableness scales are considered more easy-going, friendly, and trusting compared to those with lower Agreeableness (Costa and McCrae, 1980; Kang et al., 2023). This trait is also positively related to the use of emotion regulation strategies which emphasise improving an emotional state (Connor-Smith and Flachsbart, 2007; Baranczuk, 2019). Based on this, our findings support the perspective that individuals who are more agreeable have stronger desire to feel better as opposed to worse.

#### 4.2.2. Conscientiousness

Individuals who are highly conscientious are considered to be self-disciplined, mindful, persistent, and less prone to experiencing mental health problems (Kotov et al., 2010; Rettew et al., 2021). Baranczuk (2019) suggests that these qualities may be associated with typically adaptive emotion regulation strategies (such as problem solving and reappraisal), which are themselves associated with greater positive affect (Augustine et al., 2010). It is possible that more conscientious individuals may have greater desire for general positive affect. However, we found no evidence that this trait predicted musicians’ desire to regulate any practice-related emotions. One explanation for this finding is that an emotional state of *any* kind may not be helpful for an individual with conscientious tendencies (Costa and McCrae, 1992). An alternative explanation is that more conscientious musicians did not feel a need to regulate these emotions as their *actual* emotions were already at a sufficient intensity.

#### 4.2.3. Emotional Stability (*inverse Neuroticism*)

Individuals with higher emotional stability have greater resilience to stress and experience less negative affect compared to individuals with lower emotional stability (Kotov et al., 2010; Verduyn and Brans, 2012; Mitchell et al., 2021). Neuroticism itself is positively associated with worse mental health (e.g., Kang, 2023) and accordingly, individuals who are highly neurotic (i.e., with lower emotional stability) experience greater levels of negative affect (Barrick et al., 2021). It is possible then that individuals with higher Emotional Stability scores may experience and desire less unpleasant affect in their musical practice. Interestingly however, higher scores for this trait predicted less desire to decrease the intensity of unpleasant emotions, coupled with less desire to increase positive ones. We suggest two possible explanations for these findings. First, Emotional Stability is a trait rooted in avoidance and inhibitory behaviours (Miles and Hempel, 2003). More emotionally stable individuals may seek to maintain an existing emotional state rather than enhancing or reducing the intensity of their emotions. An alternative explanation is that these individuals may simply be emotionally stable enough that their experienced emotions do not represent a barrier for them, and do not feel the need to change them in a musical practice situation.

#### 4.2.4. Extraversion

Extraversion is associated with greater interest in social interactions and a stronger tendency to experience positive and highly activated emotions compared to more introverted individuals (Kampfe and Mitte, 2009; Augustine et al., 2010; Verduyn and Brans, 2012; Kang et al., 2023). This trait is also associated with greater expression of emotions, including anger (King and Emmons, 1990; Martin et al., 1999). This quality of Extraversion may play a role in the context of musical practice, as a higher score for this trait was associated with greater desire to increase the intensity of anger. Although anger may sometimes be maladaptive (e.g., if it leads to violence), it can also be motivational (Ellsworth and Smith, 1988) and may help an individual to be more assertive (Tamir, 2016; Ortner et al., 2018). In the context of musical practice, more extraverted musicians may desire to up-regulate anger in order to exploit the motivational properties of this emotion. The maladaptive qualities of anger may then be avoided in musical practice if anger is experienced in conjunction with stronger, pleasant emotions (see Table 2). Targeting a mixed affective state (both positive and negative emotions together) is a strategy associated with stronger musical practice goals related to Mastery (Breaden Madden and Jabusch, 2021).

#### 4.2.5. Openness

Individuals who score highly on Openness scales are characterised as creative and imaginative, with a greater tendency to experience a wide range of emotions compared to those with lower scores in this trait (McCrae, 2007; Baranczuk, 2019). Our findings align with this information; we found that Openness predicted the desire to increase and decrease a combination of both positive and negative emotions. Openness is positively associated with the diverse usage of both adaptive and maladaptive emotion regulation strategies (Connor-Smith and Flachsbart, 2007). Whilst adaptive strategies tend to emphasise positive emotions, maladaptive strategies tend to involve unpleasant emotions. This may account for why higher Openness predicted less desire to reduce unpleasant emotions. Furthermore, the tendency to retain emotions is one defining characteristic of Openness (Baranczuk, 2019). It is possible that highly open individuals may avoid emotion regulation behaviours which suppress any emotional experience; these individuals value having emotional experiences even if they are unpleasant.

### 4.3. Mastery-related DES and the desire for trait-consistent affect

Mastery goal orientation impacted the existing relationships between personality traits and emotions in three different ways:

#### 4.3.1. Amplifying effects

In two cases, the initial relationship between a trait and an emotion was amplified in strength. When a musician was high in Agreeableness there was a stronger desire for focus/concentration, but when Mastery orientation was also at a high level, there was *even stronger* desire to increase it. This was also the case for Emotional Stability. When a musician was high in this trait there was a stronger desire for happiness, but when Mastery orientation was also high, there was an even stronger desire to increase this particular emotion.

#### 4.3.2. Mitigating/Reversing effects

Several instances were identified where Mastery goal orientation mitigated or even reversed the initial trait-emotion relationship. For example, higher levels of Agreeableness initially predicted less desire to increase gloom. However, when Mastery was at a moderately high level (∼1 SD above the mean) this effect was mitigated—and at higher levels (e.g., 2 SD above the mean) even reversed. In addition, when a musician was high in Openness, there was less desire to reduce the intensity of gloom. But when Mastery orientation was also at a higher level, there was instead greater desire to reduce it.

#### 4.3.3. Introducing new effects

Mastery goal orientation also introduced a range of new effects which were not identified in the initial *Emotion Increase* or *Emotion Decrease* models. For example, higher Conscientiousness did not initially predict the desire to regulate any of the assessed emotions. However, as Mastery orientation increased, this trait became predictive of a reduced desire to increase several pleasant emotions. While Extraversion did not initially predict the desire to reduce anger, the inclusion of Mastery orientation revealed that Extraversion increasingly predicted less strong desire to reduce it. Finally, Openness initially had no impact on musicians’ desire to regulate low-activated unpleasant emotions whereas with higher levels of Mastery orientation, Openness increasingly predicted greater desire to reduce several low-arousal unpleasant emotions including downheartedness and gloom.

The above three impacts (*amplification*, *mitigation/reversal*, and *introduction of new effects*) frame Mastery goal orientation as an influential aspect of high-performance, deliberate emotion regulation in musical practice. Developing musical mastery requires significant conscious effort to improve one’s skills, adapting to changing circumstances, and the capacity to persevere in spite of challenges. Successful music performers are able to eliminate unwanted emotions that may interfere with their musical activities whilst at the same time seeking an emotional state which facilitates performance at a high level (Woody and McPherson, 2010). Musical practice may not be different in this respect. Although our findings in general do not undermine a global hedonic principle (after all, in many cases musicians *did* desire positive emotions and less negative ones), we did not identify any trait-related blanket effects. In other words, traits did not modulate musicians’ desire to regulate either *all* positive or *all* negative emotions. Instead, when musicians indicated strong Mastery goal orientation, their personality traits aligned to a specific subset of emotions – often a combination of both positive and negative. This suggests that, as far as musical practice is concerned, the DES may transcend a singular principle whereby musicians seek only to improve an emotional state (i.e., increasing positive emotions and reducing negative ones). In that sense, musicians who strive for effective practice as part of a Mastery goal orientation may engage in a highly selective, “cherry-picking” emotion regulation process in order to bring about whichever combination of emotions may facilitate better practice. This contrasts with the premise that high-performance activities are associated with (and sometimes supposedly dependent on) exclusively positive affective states (see Lane, 2012). For instance, we identified cases where musicians indicated less desire to increase pleasant emotions as well as greater desire to increase unpleasant emotions such as anger. Take for example, a hypothetical musician who does not desire to experience greater levels of happiness. The most straightforward explanation for this desire is that happiness is already experienced at a sufficient intensity and this musician does not feel the need to change it, merely to sustain it. Importantly however, less desire to increase a positive emotion does *not* indicate a greater desire to reduce it (desire to up-regulate and down-regulate not necessarily being opposites on the same spectrum), nor does it indicate a desire to supplant, for example, happiness with other emotions). With this in mind, our findings complement recent work from Breaden Madden and Jabusch (2021). In that study, musicians with strong mastery orientation sought an emotional state consisting of up-regulated pleasant emotions combined with moderately up-regulated anger and nervousness. The authors argue that this “mixed” emotional state may be a regulatory tendency which has developed with greater exposure to the challenges of musical mastery.

Given the diverse impact of Mastery goal orientation on musical practice-related DES, it is possible that the desire for specific emotions may to some extent be driven by musicians’ preference for trait-consistent affect when under challenging goal conditions. We highlight two examples below which may indicate such a desire for trait-consistent affect, at a descriptive level. First, with higher levels of Mastery orientation, Extraversion increasingly predicted less strong desire to decrease anger. Highly extraverted individuals experience greater levels of highly activated emotions in general, regardless of whether they are positive or negative (Augustine et al., 2010). Less desire to reduce anger is trait-consistent with the qualities of extraversion, especially if it is already experienced at sufficient intensity. Second, our analysis indicated that when a musician was high in Emotional Stability, there was less desire for happiness, but when Mastery orientation was at a high level, there was even *less* desire to increase this emotion. This finding may have arisen as happiness was *actually* experienced at a high level. However, under challenging performance conditions, individuals who score higher (vs. lower) in Neuroticism desire trait-consistent unpleasant emotions (Tamir, 2005; Leung et al., 2014). Therefore, individuals who are less neurotic (i.e., scoring higher in Emotional Stability) may instead desire trait-consistent pleasant affect under similar conditions (such as the challenging conditions of Mastery-oriented musical practice). In that sense maintaining stronger levels of happiness under challenging Mastery goal conditions may indicate a desire to sustain trait-consistent positive affect.

### 4.4. Implications for musicians’ health and goal-related practice

Emotion regulation literature often emphasises the benefit of reducing unpleasant emotions. This is of course a reasonable emphasis, as excessive experience of unpleasant emotions can negatively impact mental health and lead to problems such as depression and anxiety (Tamir et al., 2008). Active engagement with music is known to involve both positive and negative emotions (Hallam, 2010) and it is generally accepted that these negative emotions may be detrimental (e.g., fear that leads to avoidance of learning). Our findings indicate that the unpleasant emotions *actually* experienced by musicians in their practice were experienced at a relatively low level, and the *desired* emotions were generally strongly positive. This is an important finding from a health and wellbeing perspective. It suggests that potentially harmful unpleasant emotions do not feature strongly in musicians’ practice. Importantly, we found that musicians, regardless of the level of any personality trait, had no desire to increase anxiety. Experiencing anxiety is well understood to be detrimental to individuals’ wellbeing and health, and may occur specifically within the context of musical practice. For example, in their study on anxiety, practice behaviours and music performance quality, Passarotto et al. (2023) showed positive associations between several measures of self-reported anxiety and practice time, in addition to similar positive correlations between anxiety and the quantity of repetitive practice behaviours. Furthermore, Performance Anxiety (Kenny, 2011) is a serious and debilitating condition which can negatively impact the life of a performing musician, sometimes even discouraging them from pursuing further musical activities when experienced at a sufficiently high level. Highlighting low reported levels of experienced and desired anxiety in musical practice is additionally relevant given the large contingent of student musicians in our sample (71%). At first view this finding contrasts with that of Spahn et al. (2004) who reported elevated anxiety among music students compared to students in other disciplines: In their sample, 33.5% of music students reported anxiety levels that were in the borderline or elevated range. This discrepancy may be explained by the fact that Spahn et al. (2004) assessed self-evaluated anxiety within the last week preceding the completion of their questionnaire whereas the focus of our study was experienced and regulated emotions (including anxiety) in musical practice only. Emotional states in musical practice may be different compared to those in non-musical, everyday situations. One example is the so-called flow state (Csikszentmihályi, 1990; Araújo and Hein, 2019) which may be experienced by musicians in practice (as well as in performance) and which is associated with positive (and less negative) emotions. This may specifically apply to the experience of anxiety and may partially explain the finding of low reported levels of experienced and regulated anxiety in our study. Indeed, within this sample, greater levels of experienced anxiety was negatively correlated with several flow-state items (e.g., “I am totally focussed on what I am doing in practice”—Spearman’s ρ = −0.241; *p* < 0.001; “I really enjoy the experience of musical practice”—Spearman’s ρ = −0.276, *p* < 0.001. Please see Breaden Madden and Jabusch (2021) for a complete description of flow-related items used in this study). Furthermore, musicians’ desire to up-regulate other unpleasant emotions assessed here was frequently unrelated to their personality traits. This contrasts with existing literature that often associates Neuroticism with greater tendency to experience negative affect (e.g., Verduyn and Brans, 2012), and helps to rebut the stereotype that neurotic individuals always want to feel negative (Rusting and Larsen, 1995). That the desire to experience a broad range of unpleasant emotions was unrelated to personality traits is an encouraging aspect of our findings. It suggests that musicians who have a particularly strong personality disposition (e.g., low Emotional Stability) may not be at greater risk of experiencing potentially harmful unpleasant emotions in their practice. We suggest that those rare cases in which a musician desires to intensify an unpleasant emotion during their practice does not indicate that they want musical practice to be an unpleasant experience. Rather, up-regulating unpleasant affect can be understood as a potential desire to find optimum arousal for optimum performance (Yerkes and Dodson, 1908; Lehmann et al., 2007; Woody and McPherson, 2010). Presumably then, selecting an unpleasant emotion in the short-term may take place in the hope of producing success in the long-term. Particularly when pursuing a very challenging goal (such as mastery), this in itself should be a positive experience (Tamir, 2009; Lane et al., 2011). After all, Breaden Madden and Jabusch (2021) showed that the majority of musicians who were older (vs. younger) and identified as a professional (vs. student) selected a *mixed* emotional state (consisting of up-regulated positive and negative emotions together) in order to support their musical practice.

### 4.5. Strengths and limitations

There are several strengths to this study. The use of the TIPI as a standardised and well-supported measure of personality enables comparisons between the emotional experiences of musicians and individuals in other contexts as they relate to individual characteristics. Capturing desired affect via separate scales specifically for the up-regulation and down-regulation of different emotions allowed us to examine in depth the content of Mastery-related emotion states.

There are some limitations in this study. First, participants retrospectively self-reported their desire to regulate their emotions. Although retrospective self-report is considered reliable for assessing musical activities such as accumulated practice hours (Lehmann and Ericsson, 1998; Bengtsson et al., 2005), there is inherent bias in this type of data which cannot be ruled out with respect to self-reported psychological data yielded in this study. Second, the vast majority of musicians in this sample were strongly involved with Classical and Post-1950’s Contemporary Classical music styles. To the extent that our findings may have implications for health, wellbeing and individualised practice strategies, it is important to acknowledge that there may be systematic personality differences between classical musicians and musicians involved in other styles (see Kemp, 1996). Such differences may influence the emotions they desire for Mastery-related practice within their respective musical genres. Third, this study did not include a measure of progress or practice outcomes. As a result, we cannot claim any specific advantages or disadvantages for short-term or long-term musical practice success as a consequence of emotion regulation behaviour related to personality traits or Mastery goal orientation.

### 4.6. Directions for development

As musicians gain experience over time, they have more opportunities to resolve practice-related challenges and regulate their emotions in a practice environment. Future research could therefore aim to understand whether experience influences the content of a practice-related DES, and how musicians utilise different emotion regulation strategies to support specific outcomes at different stages of their musical lives. Our findings indicate which emotions musicians actually experienced and which they desired in their practice. This may broadly indicate which type(s) of emotion regulation strategies they engaged in. As regulation strategies vary in their efficacy (Augustine and Hemenover, 2009), future research could focus on understanding which strategy may best bring about different DESs, and which strategies are actually useful given a specific personality. Information of this kind may have value in other domains where high performance plays a crucial role. Chess is one example where performance at a high level is valuable (Aciego et al., 2012). Although an inherent part of elite level chess is a strong emotional stress load, research in this field tends to concentrate on intellectual rather than emotional performance (Charness et al., 2005). Finally, musicians who strive to become professionals may encounter many occupational challenges, both in- and outside the practice context. While emotion self-regulation and personal effort are necessary aspects of achievement and success, overcoming occupational challenges is not always the sole responsibility of the musician. Developing more supportive occupational and paedagogical environments may reduce musicians’ use of potentially maladaptive emotion regulation strategies. Research on this topic may also inform policies that aim to alleviate stressful occupational conditions that are relevant for musicians (such as dwindling employment opportunities) and contribute to public psychology efforts to improve health and wellbeing through lasting social change (e.g., Minde et al., 1985; Eaton et al., 2021).

## 5. Conclusion

We investigated the relationship between musicians’ personality traits, practice-related mastery goal orientation, and the emotions they desired in their musical practice. Findings indicate a broad hedonic tone underlying the emotions actually experienced by musicians in their practice. Musicians’ desired emotions suggest a highly selective, cherry-picking emotion regulation process that involves more than the up-regulation of positive emotions *as a whole* or the down-regulation of negative emotions *as a whole*. Aspects of this cherry-picking behaviour appear to be dependent on mastery goal orientation, personality, and their interaction. Understanding individual differences in the context of goal-related emotion regulation is a matter of importance as it can influence how successful our efforts are. This of course has long-term consequences for our happiness, wellbeing, and professional success when goals are consequently pursued in an effective manner.

## Data availability statement

The dataset for this article is not publicly available because public availability of data is not covered by the ethics application and the ethics approval provided for this study (Institutional Review Board of the State Chamber of Physicians of Saxony) (IRB: 425931; Application reference: EK-BR-73/19-1). Requests to access the datasets should be directed to GBM, gerard.madden@hfmdd.de. The *r* scripts used for data analysis can be viewed in the Supplementary material.

## Ethics statement

The study involved human participants and was reviewed and approved by the Institutional Review Board of the State Chamber of Physicians of Saxony (IRB: 425931; Application reference: EK-BR-73/19-1). The participants provided their written informed consent to take part in this study.

## Author contributions

GBM conceived and conducted the study, performed the statistical analysis, and wrote and reviewed the manuscript. SH performed the statistical analysis and wrote and reviewed the manuscript. SB discussed the study design and reviewed the manuscript. H-CJ discussed the study design and statistical analysis and wrote and reviewed the manuscript. All authors contributed to the article and approved the submitted version.

## Footnotes

1
https://github.com/gbmadden/EmotionalCherryPicking
