# Supplementary material for: Emotional cherry picking: the role of personality and goal orientation in selective emotion regulation for musical practice
Source: Front Psychol. 2023 Jul 27;14:1201442. doi: 10.3389/fpsyg.2023.1201442 (PMC10415679; doi:10.3389/fpsyg.2023.1201442)
Supplement: Supplementary file 1 [file Data_Sheet_1.pdf]

## Supplementary material for the article:

# Emotional cherry picking: the role of personality and goal orientation in selective emotion regulation during musical practice


Gerard Breaden Madden*, Steffen A. Herff, Scott W. Beveridge, & Hans-Christian Jabusch

*Corresponding author: gerard.madden@hfmdd.de


## Contents:

**Appendix A:** Complete fitted model for all Bayesian Mixed Effects models

To investigate main effects and interactions, we report a combination of *directed* and *exploratory* hypothesis tests. These tests evaluate the evidence of a given effect to be smaller or larger than zero (Evidence Ratio), as well as the coefficient estimates (Estimate) and the error within this estimate (Error). In the case of *directed* hypothesis tests, we consider evidence ratios ≥ 19 analogous to significant evidence in favour of an effect under an alpha level of .05. For *exploratory* hypothesis tests, we consider evidence ratios ≥ 39 analogous to significant evidence for an effect. Evidence ratios of ≥ 19 and ≥ 39 are referred to as 'strong' evidence for their respective hypotheses (Milne and Herff, 2020).

We opted to report a combination of directed and exploratory hypothesis tests in light of the different volume of research available for each specific personality trait within the existing literature. We used directed hypothesis tests for Extraversion and Emotional Stability (i.e., tests with a less conservative evidence threshold - ≥ 19; the Bayesian equivalent to a one-sided frequentist hypothesis test; Makowski et al., 2019; Marsman and Wagenmakers, 2017). We used exploratory hypothesis tests for the traits Agreeableness, Conscientiousness, and Openness (i.e., tests with a more conservative evidence threshold - ≥ 39; the Bayesian equivalent of a two-sided frequentist hypothesis test, Makowski et al., 2019; Marsman and Wagenmakers, 2017). The chosen prior, scaling, and evidence reference are commonly used in the music cognition and perception literature (Cecchetti et al., 2021; Dobrowohl et al., 2019; Herff et al., 2020a, 2020b, Herff et al., 2021a, 2021b; Jääskeläinen et al., 2020; MacRitchie et al., 2020; Smit et al., 2020; Smit et al., 2021).

Tables A1 and A2 in Appendix A contain for all emotions the estimated mean of the standardised effect (*Estimate*), the standard error of the estimated mean (*Est. Error*) and evidence radio in favour of a hypothesis (*Odds (< or > 0))*. Within the main text, only estimates with an odds ratio ≥ 19 (for Extraversion and Emotional Stability) and ≥ 39 (for Agreeableness, Conscientiousness, Openness) are reported. For further information, please see the *Statistical Approach* section of the article.

**Appendix B:** *R* script for all Bayesian Mixed Effects models

Appendix B provides the complete *R* script for running the Bayesian models.
The script includes the setup (package installation, variable scaling) and specification for each of the four models.

**Appendix C:** *R* script for all Bayesian hypothesis tests

Appendix B provides the complete *R* script for running the hypothesis tests for each of the four models.

**References**

# Appendix A

**A1.** Summary of directional and exploratory hypothesis tests for the effects of the *Emotion Increase* and *Emotion Decrease* models.

| | | Personality Trait | | | | | | | | | |
|---|---|---|---|---|---|---|---|---|---|---|---|
| | | **Agreeableness** | | **Conscientiousness** | | **Emotional Stability** | | **Extraversion** | | **Openness** | |
| | | **Increase** | **Decrease** | **Increase** | **Decrease** | **Increase** | **Decrease** | **Increase** | **Decrease** | **Increase** | **Decrease** |
| **Anger** | *Estimate* | -0.07 | 0.01 | 0.01 | -0.03 | -0.01 | -0.05 | 0.05 | -0.06 | 0.09 | -0.02 |
| | *Est. Error* | 0.04 | 0.05 | 0.03 | 0.04 | 0.04 | 0.05 | 0.03 | 0.04 | 0.03 | 0.04 |
| | *Odds* | 30.46 | 1.55 | 1.38 | 3.13 | 1.28 | 4.33 | 43.03 | 15.18 | 2332.33 | < 9999 |
| **Anxiety** | *Estimate* | -0.01 | 0.01 | -0.05 | 0.05 | -0.02 | -0.1 | -0.01 | 0.03 | -0.02 | -0.07 |
| | *Est. Error* | 0.04 | 0.05 | 0.03 | 0.04 | 0.04 | 0.05 | 0.03 | 0.04 | 0.03 | 0.04 |
| | *Odds* | 1.48 | 1.27 | 23.33 | 11.84 | 2.43 | 36.18 | 1.39 | 3.82 | 2.48 | 25.49 |
| **Calmness** | *Estimate* | 0.1 | 0.02 | 0.02 | -0.04 | -0.17 | 0.02 | -0.03 | 0.01 | -0.03 | -0.04 |
| | *Est. Error* | 0.04 | 0.05 | 0.03 | 0.04 | 0.04 | 0.05 | 0.03 | 0.04 | 0.03 | 0.04 |
| | *Odds* | 240.38 | 2.04 | 3.32 | 5.12 | < 9999 | 1.63 | 6.57 | 1.63 | 7.82 | 6.43 |
| **Downheartedness** | *Estimate* | 0.02 | -0.08 | -0.04 | 0.05 | -0.03 | -0.04 | -0.01 | 0.04 | -0.01 | -0.04 |
| | *Est. Error* | 0.04 | 0.05 | 0.03 | 0.04 | 0.04 | 0.05 | 0.03 | 0.04 | 0.03 | 0.04 |
| | *Odds* | 2.34 | 13.78 | 21.19 | 11.6 | 3.31 | 3.12 | 0.73 | 5.76 | 1.46 | 6.89 |
| **Energy** | *Estimate* | 0.13 | 0.01 | -0.05 | -0.04 | -0.15 | 0 | 0.02 | 0 | -0.12 | -0.03 |
| | *Est. Error* | 0.04 | 0.05 | 0.03 | 0.04 | 0.04 | 0.05 | 0.03 | 0.04 | 0.03 | 0.04 |
| | *Odds* | 27999 | 1.24 | 24 | 4.85 | 27999 | 1.02 | 3.12 | 0.99 | < 9999 | 3.81 |
| **Focus / Concentration** | *Estimate* | 0.08 | 0.01 | -0.04 | -0.03 | -0.06 | 0.01 | 0.06 | 0.01 | -0.13 | -0.07 |
| | *Est. Error* | 0.04 | 0.05 | 0.03 | 4.39 | 0.04 | 0.05 | 0.03 | 0.04 | 0.03 | 0.04 |
| | *Odds* | 62.64 | 1.38 | 14.2 | 0.23 | 18.16 | 1.26 | 54.34 | 1.88 | < 9999 | 35.36 |
| **Gloom** | *Estimate* | -0.09 | 0.08 | -0.04 | 0.03 | 0.02 | -0.15 | 0 | 0.06 | 0.02 | -0.01 |
| | *Est. Error* | 0.04 | 0.05 | 0.03 | 0.04 | 0.04 | 0.05 | 0.03 | 0.04 | 0.03 | 0.04 |
| | *Odds* | 98.29 | 13.44 | 12.46 | 0.31 | 2.29 | 594.74 | 0.98 | 15.67 | 3.73 | 303.35 |
| **Guilt** | *Estimate* | 0 | -0.04 | -0.04 | 0.02 | -0.01 | -0.04 | -0.01 | 0.04 | 0 | -0.04 |
| | *Est. Error* | 0.04 | 0.05 | 0.03 | 0.04 | 0.04 | 0.05 | 0.03 | 0.04 | 0.03 | 0.04 |
| | *Odds* | 1.16 | 3.28 | 14.21 | 2.34 | 1.33 | 3.5 | 2.3 | 6.99 | 0.81 | 6.4 |
| **Happiness** | *Estimate* | 0.13 | 0.01 | -0.03 | -0.03 | -0.18 | 0 | -0.01 | -0.01 | -0.15 | -0.03 |
| | *Est. Error* | 0.04 | 0.05 | 0.03 | 0.04 | 0.04 | 0.05 | 0.03 | 0.04 | 0.03 | 0.04 |
| | *Odds* | 3499 | 1.57 | 5.73 | 4.08 | 9999 | 1.17 | 1.84 | 1.52 | < 9999 | 3.31 |
| **Nervousness** | *Estimate* | -0.01 | -0.05 | -0.03 | 0.06 | 0 | -0.05 | 0 | -0.02 | 0.02 | -0.12 |
| | *Est. Error* | 0.04 | 0.05 | 0.03 | 0.04 | 0.04 | 0.05 | 0.03 | 0.04 | 0.03 | 0.04 |
| | *Odds* | 1.77 | 4.79 | 8.33 | 21.28 | 1.02 | 5.77 | 0.87 | 2.83 | 3.97 | 1165.67 |
| **Sluggishness** | *Estimate* | 0.01 | -0.02 | -0.04 | -0.01 | -0.02 | -0.05 | -0.01 | 0.05 | 0 | -0.07 |
| | *Est. Error* | 0.04 | 0.05 | 0.03 | 0.04 | 0.04 | 0.05 | 0.03 | 0.04 | 0.03 | 0.04 |
| | *Odds* | 1.47 | 2.17 | 10.68 | 1.35 | 2.26 | 4.34 | 1.67 | 13.34 | 1.09 | 28.35 |

Grey cells show the effects related to the Emotion Increase model, and white cells refer to effects related to the Emotion Decrease model

**A2.** Summary of directional and exploratory hypothesis tests for the effects of the *Mastery Increase* and *Mastery Decrease* models.

| | | Personality Trait x Goal Interaction | | | | | | | | | |
|---|---|---|---|---|---|---|---|---|---|---|---|
| | | Agreeableness x Mastery | | Conscientiousness x Mastery | | Emotional Stability x Mastery | | Extraversion x Mastery | | Openness x Mastery | |
| | | **Increase** | **Decrease** | **Increase** | **Decrease** | **Increase** | **Decrease** | **Increase** | **Decrease** | **Increase** | **Decrease** |
| **Anger** | *Estimate* | 0.03 | -0.02 | 0.02 | -0.02 | -0.03 | 0.02 | 0.03 | -0.11 | 0.02 | -0.03 |
| | *Est. Error* | 0.04 | 0.05 | 0.03 | 0.04 | 0.04 | 0.05 | 0.03 | 0.04 | 0.03 | 0.04 |
| | *Odds* | 3.85 | 2.23 | 3.58 | 2.04 | 3.22 | 2.21 | 5.97 | 399 | 4.42 | 3.39 |
| **Anxiety** | *Estimate* | -0.05 | -0.07 | 0.03 | 0 | 0.02 | 0.08 | 0 | -0.02 | 0.01 | 0.06 |
| | *Est. Error* | 0.04 | 0.05 | 0.03 | 0.04 | 0.04 | 0.05 | 0.03 | 0.04 | 0.03 | 0.04 |
| | *Odds* | 10.05 | 11.17 | 6.89 | 1.14 | 2.22 | 15.41 | 1.07 | 2.08 | 2.22 | 23.58 |
| **Calmness** | *Estimate* | 0.03 | -0.01 | -0.05 | -0.01 | 0.02 | 0 | -0.01 | -0.03 | -0.05 | 0.02 |
| | *Est. Error* | 0.04 | 0.05 | 0.03 | 0.04 | 0.04 | 0.05 | 0.03 | 0.04 | 0.03 | 0.04 |
| | *Odds* | 4.53 | 1.19 | 33.61 | 1.67 | 2.94 | 1.17 | 1.45 | 4.19 | 63.22 | 2.43 |
| **Downheartedness** | *Estimate* | -0.03 | -0.04 | 0.03 | 0.01 | -0.01 | 0.04 | -0.02 | -0.05 | -0.03 | 0.08 |
| | *Est. Error* | 0.04 | 0.05 | 0.03 | 0.04 | 0.04 | 0.05 | 0.03 | 0.04 | 0.03 | 0.04 |
| | *Odds* | 4.02 | 3.32 | 9.5 | 1.27 | 1.34 | 3.83 | 2.55 | 11.98 | 6.35 | 65.04 |
| **Energy** | *Estimate* | 0.04 | 0.01 | -0.06 | -0.01 | -0.02 | 0 | -0.01 | -0.02 | -0.01 | -0.01 |
| | *Est. Error* | 0.04 | 0.05 | 0.03 | 0.04 | 0.04 | 0.05 | 0.03 | 0.04 | 0.03 | 0.04 |
| | *Odds* | 6.97 | 1.48 | 71.35 | 1.27 | 1.99 | 0.98 | 2.18 | 2.27 | 1.38 | 1.58 |
| **Focus / Concentration** | *Estimate* | 0.08 | -0.02 | -0.06 | -0.04 | -0.03 | 0.06 | -0.07 | 0.01 | -0.01 | 0 |
| | *Est. Error* | 0.04 | 0.05 | 0.03 | 0.04 | 0.04 | 0.05 | 0.03 | 0.04 | 0.03 | 0.04 |
| | *Odds* | 61.08 | 1.9 | 110.55 | 4.88 | 4.27 | 8.49 | 284.71 | 0.61 | 2.39 | 1.07 |
| **Gloom** | *Estimate* | 0.09 | -0.09 | -0.01 | 0.03 | -0.12 | 0.14 | -0.01 | -0.07 | -0.06 | 0.12 |
| | *Est. Error* | 0.04 | 0.05 | 0.03 | 0.04 | 0.04 | 0.05 | 0.03 | 0.04 | 0.03 | 0.04 |
| | *Odds* | 115.67 | 23.22 | 2.44 | 3.59 | 1999 | 332.33 | 1.41 | 23.93 | 98.64 | 3999 |
| **Guilt** | *Estimate* | -0.02 | -0.01 | 0.03 | 0.04 | -0.02 | 0.03 | 0.01 | -0.06 | -0.03 | 0.08 |
| | *Est. Error* | 0.04 | 0.05 | 0.03 | 0.04 | 0.04 | 0.05 | 0.03 | 0.04 | 0.03 | 0.04 |
| | *Odds* | 2.15 | 1.43 | 6 | 6.14 | 2.15 | 2.26 | 1.4 | 12.42 | 5.13 | 77.65 |
| **Happiness** | *Estimate* | 0.07 | 0 | -0.09 | -0.01 | -0.08 | 0.04 | -0.01 | -0.03 | -0.03 | 0 |
| | *Est. Error* | 0.04 | 0.05 | 0.03 | 0.04 | 0.04 | 0.05 | 0.03 | 0.04 | 0.03 | 0.04 |
| | *Odds* | 29.47 | 1 | 1865.67 | 1.73 | 94.24 | 3.33 | 9332.33 | 3.3 | 5.74 | 1.26 |
| **Nervousness** | *Estimate* | -0.03 | -0.05 | 0.04 | 0.02 | 0.02 | 0.03 | 0.01 | -0.04 | 0.01 | 0.01 |
| | *Est. Error* | 0.04 | 0.05 | 0.03 | 0.04 | 0.04 | 0.05 | 0.03 | 0.04 | 0.03 | 0.04 |
| | *Odds* | 3.23 | 4.93 | 17.83 | 2.55 | 2.78 | 2.93 | 2.11 | 4.88 | 1.42 | 1.74 |
| **Sluggishness** | *Estimate* | -0.03 | -0.03 | 0.03 | 0.01 | 0 | 0.08 | 0.01 | -0.02 | -0.01 | 0.09 |
| | *Est. Error* | 0.04 | 0.05 | 0.03 | 0.04 | 0.04 | 0.05 | 0.03 | 0.04 | 0.03 | 0.04 |
| | *Odds* | 3.3 | 2.44 | 6.6 | 1.73 | 1.08 | 17.97 | 1.37 | 2.03 | 2.35 | 160.85 |

Grey cells show the effects related to the Mastery Increase model, and white cells refer to effects related to the Mastery Decrease model

## Appendix B.

*brms* script for Bayesian Mixed Effects Models. B.1) *Emotion Increase* model; B.2) *Emotion Decrease* model; B3). *Mastery Increase* model; B4) *Mastery Decrease* model

```
# Emotional cherry picking: the role of personality and goal orientation in selective emotion regulation during musical practice
# AUTHORS: Gerard Breaden Madden, Steffen A. Herff, Scott W. Beveridge, Hans-Christian Jabusch
# CORRESPONDING AUTHOR: gerard.madden@hfmdd.de
```

```
# SETUP FOR ALL MODELS

install.packages("readxl")
install.packages("devtools")
install.packages("brms")
install.packages("rstanarm")

library("readxl")
library("devtools")
library("brms")
library("rstanarm")

options(mc.cores = parallel::detectCores())

bayes_data <- read_excel("DATASET DIRECTORY & EXCEL FILE NAME ")

# SCALING FOR ALL MODELS

bayes_data$Increase_scaled <- scale(bayes_data$Increase)
bayes_data$Decrease_scaled <- scale(bayes_data$Decrease)
bayes_data$Extraversion_scaled <- scale(bayes_data$Extraversion)
bayes_data$Agreeableness_scaled <- scale(bayes_data$Agreeableness)
bayes_data$Conscientiousness_scaled <- scale(bayes_data$Conscientiousness)
bayes_data$Openness_scaled <- scale(bayes_data$Openness)
bayes_data$Stability_scaled <- scale(bayes_data$Stability)
bayes_data$Mastery_scaled <- scale(bayes_data$Mastery_Goal)

# SET BASELINE

bayes_data$Emotion <- as.factor(bayes_data$Emotion)
bayes_data$Emotion <- relevel(bayes_data$Emotion, ref='Happiness')
```

**# MODEL B.1 “Emotion Increase”**

**# SET MODEL PRIORS**

```
Priors <- get_prior(Increase_scaled ~
          Extraversion_scaled*Emotion +
          Agreeableness_scaled*Emotion +
          Conscientiousness_scaled*Emotion +
          Openness_scaled*Emotion +
          Stability_scaled*Emotion + Mastery_scaled*Emotion +
         (1 | Participant),
         bayes_data,
         gaussian(link='identity') )
Priors[Priors$prior == "student_t(3, 0, 10)",'prior'] <- "student_t(3, 0, 1)"
```

**# RUN “Emotion Increase” MODEL**

```
fit_emotionincrease <- brm(Increase_scaled ~
                  Extraversion_scaled*Emotion +
                  Agreeableness_scaled*Emotion +
                  Conscientiousness_scaled*Emotion +
                  Openness_scaled*Emotion +
                  Stability_scaled*Emotion + Mastery_scaled*Emotion +
                 (1 | Participant),
                 prior= Priors,
                 family = gaussian(link='identity'),
                 warmup = 3000,
                 iter = 10000,
                 inits = 0,
                 chains = 4,
                 cores = 4,
                 control = list(adapt_delta = 0.95, max_treedepth = 15),
                 data = bayes_data)
summary(fit_emotionincrease)

conditional_effects(fit_emotionincrease)
```

**# MODEL B.2 “Emotion Decrease”**

**# SET MODEL PRIORS**

```
Priors <- get_prior(Decrease_scaled ~
          Extraversion_scaled*Emotion +
          Agreeableness_scaled*Emotion +
          Conscientiousness_scaled*Emotion +
          Openness_scaled*Emotion +
          Stability_scaled*Emotion +
          (1 | Participant),
         bayes_data,
         gaussian(link='identity') )
Priors[Priors$prior == "student_t(3, 0, 10)",'prior'] <- "student_t(3, 0, 1)"
```

**# RUN “Emotion Decrease” MODEL**

```
fit_emotiondecrease <- brm(Decrease_scaled ~
                  Extraversion_scaled*Emotion +
                  Agreeableness_scaled*Emotion +
                  Conscientiousness_scaled*Emotion +
                  Openness_scaled*Emotion +
                  Stability_scaled*Emotion +
                  (1 | Participant),
                 prior= Priors,
                 family = gaussian(link='identity'),
                 warmup = 3000,
                 iter = 10000,
                 inits = 0,
                 chains = 4,
                 cores = 4,
                 control = list(adapt_delta = 0.95, max_treedepth = 15),
                 data = bayes_data)
summary(fit_emotiondecrease)

conditional_effects(fit_emotiondecrease)
```

```
# MODEL B.3 “Mastery Increase”

# SET MODEL PRIORS

Priors <- get_prior(Inecrease_scaled ~
            Extraversion_scaled*Emotion*Mastery_scaled +
            Agreeableness_scaled*Emotion*Mastery_scaled +
            Conscientiousness_scaled*Emotion*Mastery_scaled +
            Openness_scaled*Emotion*Mastery_scaled +
            Stability_scaled*Emotion*Mastery_scaled +
            (1 | Participant),
           bayes_data,
           gaussian(link='identity') )
Priors[Priors$prior == "student_t(3, 0, 10)",'prior'] <- "student_t(3, 0, 1)"

# RUN “Mastery Increase” MODEL

fit_masteryincrease <- brm(Increase_scaled ~
                   Extraversion_scaled*Emotion*Mastery_scaled +
                   Agreeableness_scaled*Emotion*Mastery_scaled +
                   Conscientiousness_scaled*Emotion*Mastery_scaled +
                   Openness_scaled*Emotion*Mastery_scaled +
                   Stability_scaled*Emotion*Mastery_scaled +
                   (1 | Participant),
                  prior= Priors,
                  family = gaussian(link='identity'),
                  warmup = 3000,
                  iter = 10000,
                  inits = 0,
                  chains = 4,
                  cores = 4,
                  control = list(adapt_delta = 0.95, max_treedepth = 15),
                  data = bayes_data)
summary(fit_masteryincrease)

conditional_effects(fit_masteryincrease)
```

```
# MODEL B.4 “Mastery Decrease”

# SET MODEL PRIORS

Priors <- get_prior(Decrease_scaled ~
            Extraversion_scaled*Emotion*Mastery_scaled +
            Agreeableness_scaled*Emotion*Mastery_scaled +
            Conscientiousness_scaled*Emotion*Mastery_scaled +
            Openness_scaled*Emotion*Mastery_scaled +
            Stability_scaled*Emotion*Mastery_scaled +
            (1 | Participant),
           bayes_data,
           gaussian(link='identity') )
Priors[Priors$prior == "student_t(3, 0, 10)",'prior'] <- "student_t(3, 0, 1)"

# RUN “Mastery Decrease” MODEL

fit_masterydecrease <- brm(Decrease_scaled ~
                   Extraversion_scaled*Emotion*Mastery_scaled +
                   Agreeableness_scaled*Emotion*Mastery_scaled +
                   Conscientiousness_scaled*Emotion*Mastery_scaled +
                   Openness_scaled*Emotion*Mastery_scaled +
                   Stability_scaled*Emotion*Mastery_scaled +
                   (1 | Participant),
                  prior= Priors,
                  family = gaussian(link='identity'),
                  warmup = 3000,
                  iter = 10000,
                  inits = 0,
                  chains = 4,
                  cores = 4,
                  control = list(adapt_delta = 0.95, max_treedepth = 15),
                  data = bayes_data)
summary(fit_ masterydecrease)

conditional_effects(fit_masterydecrease)
```

## Appendix C.

Hypothesis Test Scripts for C.1) *Emotion Increase* model; C.2) *Emotion Decrease* model; C3). *Mastery Increase* model; C4) *Mastery Decrease* model

```
# C.1 HYPOTHESIS TESTING FOR THE EMOTION INCREASE MODEL

# AGREEABLENESS
hypothesis(fit_emotionincrease, c('Agreeableness_scaled > 0'))
hypothesis(fit_emotionincrease, c('Agreeableness_scaled + EmotionEnergy:Agreeableness_scaled > 0'))
hypothesis(fit_emotionincrease, c('Agreeableness_scaled + EmotionCalmness:Agreeableness_scaled > 0'))
hypothesis(fit_emotionincrease, c('Agreeableness_scaled + EmotionGloom:Agreeableness_scaled < 0'))
hypothesis(fit_emotionincrease, c('Agreeableness_scaled + EmotionGuilt:Agreeableness_scaled > 0'))
hypothesis(fit_emotionincrease, c('Agreeableness_scaled + EmotionSluggish:Agreeableness_scaled > 0'))
hypothesis(fit_emotionincrease, c('Agreeableness_scaled + EmotionAnxious:Agreeableness_scaled < 0'))
hypothesis(fit_emotionincrease, c('Agreeableness_scaled + EmotionDownhearted:Agreeableness_scaled > 0'))
hypothesis(fit_emotionincrease, c('Agreeableness_scaled + EmotionNervous:Agreeableness_scaled < 0'))
hypothesis(fit_emotionincrease, c('Agreeableness_scaled + EmotionAnger:Agreeableness_scaled < 0'))
hypothesis(fit_emotionincrease, c('Agreeableness_scaled + EmotionFocus:Agreeableness_scaled > 0'))

# CONSCIENTIOUSNESS
hypothesis(fit_emotionincrease, c('Conscientiousness_scaled < 0'))
hypothesis(fit_emotionincrease, c('Conscientiousness_scaled + EmotionEnergy:Conscientiousness_scaled < 0'))
hypothesis(fit_emotionincrease, c('Conscientiousness_scaled + EmotionCalmness:Conscientiousness_scaled > 0'))
hypothesis(fit_emotionincrease, c('Conscientiousness_scaled + EmotionGloom:Conscientiousness_scaled < 0'))
hypothesis(fit_emotionincrease, c('Conscientiousness_scaled + EmotionGuilt:Conscientiousness_scaled < 0'))
hypothesis(fit_emotionincrease, c('Conscientiousness_scaled + EmotionSluggish:Conscientiousness_scaled < 0'))
hypothesis(fit_emotionincrease, c('Conscientiousness_scaled + EmotionAnxious:Conscientiousness_scaled < 0'))
hypothesis(fit_emotionincrease, c('Conscientiousness_scaled + EmotionDownhearted:Conscientiousness_scaled < 0'))
hypothesis(fit_emotionincrease, c('Conscientiousness_scaled + EmotionNervous:Conscientiousness_scaled < 0'))
hypothesis(fit_emotionincrease, c('Conscientiousness_scaled + EmotionAnger:Conscientiousness_scaled > 0'))
hypothesis(fit_emotionincrease, c('Conscientiousness_scaled + EmotionFocus:Conscientiousness_scaled < 0'))

# EMOTIONAL STABILITY
hypothesis(fit_emotionincrease, c('Stability_scaled < 0'))
hypothesis(fit_emotionincrease, c('Stability_scaled + EmotionEnergy:Stability_scaled < 0'))
hypothesis(fit_emotionincrease, c('Stability_scaled + EmotionCalmness:Stability_scaled < 0'))
hypothesis(fit_emotionincrease, c('Stability_scaled + EmotionGloom:Stability_scaled > 0'))
hypothesis(fit_emotionincrease, c('Stability_scaled + EmotionGuilt:Stability_scaled < 0'))
hypothesis(fit_emotionincrease, c('Stability_scaled + EmotionSluggish:Stability_scaled < 0'))
hypothesis(fit_emotionincrease, c('Stability_scaled + EmotionAnxious:Stability_scaled < 0'))
hypothesis(fit_emotionincrease, c('Stability_scaled + EmotionDownhearted:Stability_scaled < 0'))
hypothesis(fit_emotionincrease, c('Stability_scaled + EmotionNervous:Stability_scaled > 0'))
hypothesis(fit_emotionincrease, c('Stability_scaled + EmotionAnger:Stability_scaled < 0'))
hypothesis(fit_emotionincrease, c('Stability_scaled + EmotionFocus:Stability_scaled < 0'))
```

```r
# EXTRAVERSION
hypothesis(fit_emotionincrease, c('Extraversion_scaled < 0'))
hypothesis(fit_emotionincrease, c('Extraversion_scaled + Extraversion_scaled:EmotionEnergy > 0'))
hypothesis(fit_emotionincrease, c('Extraversion_scaled + Extraversion_scaled:EmotionCalmness < 0'))
hypothesis(fit_emotionincrease, c('Extraversion_scaled + Extraversion_scaled:EmotionGloom > 0'))
hypothesis(fit_emotionincrease, c('Extraversion_scaled + Extraversion_scaled:EmotionGuilt < 0'))
hypothesis(fit_emotionincrease, c('Extraversion_scaled + Extraversion_scaled:EmotionSluggish < 0'))
hypothesis(fit_emotionincrease, c('Extraversion_scaled + Extraversion_scaled:EmotionAnxious < 0'))
hypothesis(fit_emotionincrease, c('Extraversion_scaled + Extraversion_scaled:EmotionDownhearted < 0'))
hypothesis(fit_emotionincrease, c('Extraversion_scaled + Extraversion_scaled:EmotionNervous > 0'))
hypothesis(fit_emotionincrease, c('Extraversion_scaled + Extraversion_scaled:EmotionAnger > 0'))
hypothesis(fit_emotionincrease, c('Extraversion_scaled + Extraversion_scaled:EmotionFocus > 0'))

#OPENNESS
hypothesis(fit_emotionincrease, c('Openness_scaled < 0'))
hypothesis(fit_emotionincrease, c('Openness_scaled + EmotionEnergy:Openness_scaled < 0'))
hypothesis(fit_emotionincrease, c('Openness_scaled + EmotionCalmness:Openness_scaled < 0'))
hypothesis(fit_emotionincrease, c('Openness_scaled + EmotionGloom:Openness_scaled > 0'))
hypothesis(fit_emotionincrease, c('Openness_scaled + EmotionGuilt:Openness_scaled > 0'))
hypothesis(fit_emotionincrease, c('Openness_scaled + EmotionSluggish:Openness_scaled > 0'))
hypothesis(fit_emotionincrease, c('Openness_scaled + EmotionAnxious:Openness_scaled < 0'))
hypothesis(fit_emotionincrease, c('Openness_scaled + EmotionDownhearted:Openness_scaled < 0'))
hypothesis(fit_emotionincrease, c('Openness_scaled + EmotionNervous:Openness_scaled > 0'))
hypothesis(fit_emotionincrease, c('Openness_scaled + EmotionAnger:Openness_scaled > 0'))
hypothesis(fit_emotionincrease, c('Openness_scaled + EmotionFocus:Openness_scaled < 0'))

# MASTERY GOAL ORIENTATION
hypothesis(fit_emotionincrease, c('Mastery_scaled < 0'))
hypothesis(fit_emotionincrease, c('Mastery_scaled + EmotionEnergy:Mastery_scaled < 0'))
hypothesis(fit_emotionincrease, c('Mastery_scaled + EmotionCalmness:Mastery_scaled > 0'))
hypothesis(fit_emotionincrease, c('Mastery_scaled + EmotionGloom:Mastery_scaled < 0'))
hypothesis(fit_emotionincrease, c('Mastery_scaled + EmotionGuilt:Mastery_scaled < 0'))
hypothesis(fit_emotionincrease, c('Mastery_scaled + EmotionSluggish:Mastery_scaled < 0'))
hypothesis(fit_emotionincrease, c('Mastery_scaled + EmotionAnxious:Mastery_scaled < 0'))
hypothesis(fit_emotionincrease, c('Mastery_scaled + EmotionDownhearted:Mastery_scaled < 0'))
hypothesis(fit_emotionincrease, c('Mastery_scaled + EmotionNervous:Mastery_scaled > 0'))
hypothesis(fit_emotionincrease, c('Mastery_scaled + EmotionAnger:Mastery_scaled > 0'))
hypothesis(fit_emotionincrease, c('Mastery_scaled + EmotionFocus:Mastery_scaled > 0'))
```

```
# C.2 HYPOTHESIS TESTING FOR THE EMOTION DECREASE MODEL

# AGREEABLENESS
hypothesis(fit_emotiondecrease, c('Agreeableness_scaled > 0'))
hypothesis(fit_emotiondecrease, c('Agreeableness_scaled + EmotionEnergy:Agreeableness_scaled > 0'))
hypothesis(fit_emotiondecrease, c('Agreeableness_scaled + EmotionCalmness:Agreeableness_scaled > 0'))
hypothesis(fit_emotiondecrease, c('Agreeableness_scaled + EmotionGloom:Agreeableness_scaled > 0'))
hypothesis(fit_emotiondecrease, c('Agreeableness_scaled + EmotionGuilt:Agreeableness_scaled < 0'))
hypothesis(fit_emotiondecrease, c('Agreeableness_scaled + EmotionSluggish:Agreeableness_scaled < 0'))
hypothesis(fit_emotiondecrease, c('Agreeableness_scaled + EmotionAnxious:Agreeableness_scaled > 0'))
hypothesis(fit_emotiondecrease, c('Agreeableness_scaled + EmotionDownhearted:Agreeableness_scaled < 0'))
hypothesis(fit_emotiondecrease, c('Agreeableness_scaled + EmotionNervous:Agreeableness_scaled < 0'))
hypothesis(fit_emotiondecrease, c('Agreeableness_scaled + EmotionAnger:Agreeableness_scaled > 0'))
hypothesis(fit_emotiondecrease, c('Agreeableness_scaled + EmotionFocus:Agreeableness_scaled > 0'))

# CONSCIENTIOUSNESS
hypothesis(fit_emotiondecrease, c('Conscientiousness_scaled < 0'))
hypothesis(fit_emotiondecrease, c('Conscientiousness_scaled + EmotionEnergy:Conscientiousness_scaled < 0'))
hypothesis(fit_emotiondecrease, c('Conscientiousness_scaled + EmotionCalmness:Conscientiousness_scaled < 0'))
hypothesis(fit_emotiondecrease, c('Conscientiousness_scaled + EmotionGloom:Conscientiousness_scaled < 0'))
hypothesis(fit_emotiondecrease, c('Conscientiousness_scaled + EmotionGuilt:Conscientiousness_scaled > 0'))
hypothesis(fit_emotiondecrease, c('Conscientiousness_scaled + EmotionSluggish:Conscientiousness_scaled < 0'))
hypothesis(fit_emotiondecrease, c('Conscientiousness_scaled + EmotionAnxious:Conscientiousness_scaled > 0'))
hypothesis(fit_emotiondecrease, c('Conscientiousness_scaled + EmotionDownhearted:Conscientiousness_scaled > 0'))
hypothesis(fit_emotiondecrease, c('Conscientiousness_scaled + EmotionNervous:Conscientiousness_scaled > 0'))
hypothesis(fit_emotiondecrease, c('Conscientiousness_scaled + EmotionAnger:Conscientiousness_scaled < 0'))
hypothesis(fit_emotiondecrease, c('Conscientiousness_scaled + EmotionFocus:Conscientiousness_scaled < 0'))

# EMOTIONAL STABILITY
hypothesis(fit_emotiondecrease, c('Stability_scaled > 0'))
hypothesis(fit_emotiondecrease, c('Stability_scaled + EmotionEnergy:Stability_scaled > 0'))
hypothesis(fit_emotiondecrease, c('Stability_scaled + EmotionCalmness:Stability_scaled > 0'))
hypothesis(fit_emotiondecrease, c('Stability_scaled + EmotionGloom:Stability_scaled < 0'))
hypothesis(fit_emotiondecrease, c('Stability_scaled + EmotionGuilt:Stability_scaled < 0'))
hypothesis(fit_emotiondecrease, c('Stability_scaled + EmotionSluggish:Stability_scaled < 0'))
hypothesis(fit_emotiondecrease, c('Stability_scaled + EmotionAnxious:Stability_scaled < 0'))
hypothesis(fit_emotiondecrease, c('Stability_scaled + EmotionDownhearted:Stability_scaled < 0'))
hypothesis(fit_emotiondecrease, c('Stability_scaled + EmotionNervous:Stability_scaled < 0'))
hypothesis(fit_emotiondecrease, c('Stability_scaled + EmotionAnger:Stability_scaled < 0'))
hypothesis(fit_emotiondecrease, c('Stability_scaled + EmotionFocus:Stability_scaled > 0'))
```

```
# EXTRAVERSION
hypothesis(fit_emotiondecrease, c('Extraversion_scaled < 0'))
hypothesis(fit_emotiondecrease, c('Extraversion_scaled + Extraversion_scaled:EmotionEnergy > 0'))
hypothesis(fit_emotiondecrease, c('Extraversion_scaled + Extraversion_scaled:EmotionCalmness > 0'))
hypothesis(fit_emotiondecrease, c('Extraversion_scaled + Extraversion_scaled:EmotionGloom > 0'))
hypothesis(fit_emotiondecrease, c('Extraversion_scaled + Extraversion_scaled:EmotionGuilt > 0'))
hypothesis(fit_emotiondecrease, c('Extraversion_scaled + Extraversion_scaled:EmotionSluggish > 0'))
hypothesis(fit_emotiondecrease, c('Extraversion_scaled + Extraversion_scaled:EmotionAnxious > 0'))
hypothesis(fit_emotiondecrease, c('Extraversion_scaled + Extraversion_scaled:EmotionDownhearted > 0'))
hypothesis(fit_emotiondecrease, c('Extraversion_scaled + Extraversion_scaled:EmotionNervous < 0'))
hypothesis(fit_emotiondecrease, c('Extraversion_scaled + Extraversion_scaled:EmotionAnger < 0'))
hypothesis(fit_emotiondecrease, c('Extraversion_scaled + Extraversion_scaled:EmotionFocus > 0'))

# OPENNESS
hypothesis(fit_emotiondecrease, c('Openness_scaled < 0'))
hypothesis(fit_emotiondecrease, c('Openness_scaled + EmotionEnergy:Openness_scaled < 0'))
hypothesis(fit_emotiondecrease, c('Openness_scaled + EmotionCalmness:Openness_scaled < 0'))
hypothesis(fit_emotiondecrease, c('Openness_scaled + EmotionGloom:Openness_scaled < 0'))
hypothesis(fit_emotiondecrease, c('Openness_scaled + EmotionGuilt:Openness_scaled < 0'))
hypothesis(fit_emotiondecrease, c('Openness_scaled + EmotionSluggish:Openness_scaled < 0'))
hypothesis(fit_emotiondecrease, c('Openness_scaled + EmotionAnxious:Openness_scaled < 0'))
hypothesis(fit_emotiondecrease, c('Openness_scaled + EmotionDownhearted:Openness_scaled < 0'))
hypothesis(fit_emotiondecrease, c('Openness_scaled + EmotionNervous:Openness_scaled < 0'))
hypothesis(fit_emotiondecrease, c('Openness_scaled + EmotionAnger:Openness_scaled < 0'))
hypothesis(fit_emotiondecrease, c('Openness_scaled + EmotionFocus:Openness_scaled < 0'))

# MASTERY GOAL ORIENTATION
hypothesis(fit_emotiondecrease, c('Mastery_scaled > 0'))
hypothesis(fit_emotiondecrease, c('Mastery_scaled + EmotionEnergy:Mastery_scaled > 0'))
hypothesis(fit_emotiondecrease, c('Mastery_scaled + EmotionCalmness:Mastery_scaled < 0'))
hypothesis(fit_emotiondecrease, c('Mastery_scaled + EmotionGloom:Mastery_scaled > 0'))
hypothesis(fit_emotiondecrease, c('Mastery_scaled + EmotionGuilt:Mastery_scaled > 0'))
hypothesis(fit_emotiondecrease, c('Mastery_scaled + EmotionSluggish:Mastery_scaled > 0'))
hypothesis(fit_emotiondecrease, c('Mastery_scaled + EmotionAnxious:Mastery_scaled > 0'))
hypothesis(fit_emotiondecrease, c('Mastery_scaled + EmotionDownhearted:Mastery_scaled > 0'))
hypothesis(fit_emotiondecrease, c('Mastery_scaled + EmotionNervous:Mastery_scaled > 0'))
hypothesis(fit_emotiondecrease, c('Mastery_scaled + EmotionAnger:Mastery_scaled < 0'))
hypothesis(fit_emotiondecrease, c('Mastery_scaled + EmotionFocus:Mastery_scaled > 0'))
```

```
# C.3 HYPOTHESIS TESTING FOR THE MASTERY INCREASE MODEL

# AGREEABLENESS
hypothesis(fit_masteryincrease, c('Mastery_scaled:Agreeableness_scaled > 0'))
hypothesis(fit_masteryincrease, c('Mastery_scaled:Agreeableness_scaled + EmotionEnergy:Mastery_scaled:Agreeableness_scaled > 0'))
hypothesis(fit_masteryincrease, c('Mastery_scaled:Agreeableness_scaled + EmotionCalmness:Mastery_scaled:Agreeableness_scaled > 0'))
hypothesis(fit_masteryincrease, c('Mastery_scaled:Agreeableness_scaled + EmotionGloom:Mastery_scaled:Agreeableness_scaled > 0'))
hypothesis(fit_masteryincrease, c('Mastery_scaled:Agreeableness_scaled + EmotionGuilt:Mastery_scaled:Agreeableness_scaled < 0'))
hypothesis(fit_masteryincrease, c('Mastery_scaled:Agreeableness_scaled + EmotionSluggish:Mastery_scaled:Agreeableness_scaled  < 0'))
hypothesis(fit_masteryincrease, c('Mastery_scaled:Agreeableness_scaled + EmotionAnxious:Mastery_scaled:Agreeableness_scaled < 0'))
hypothesis(fit_masteryincrease, c('Mastery_scaled:Agreeableness_scaled + EmotionDownhearted:Mastery_scaled:Agreeableness_scaled < 0'))
hypothesis(fit_masteryincrease, c('Mastery_scaled:Agreeableness_scaled + EmotionNervous:Mastery_scaled:Agreeableness_scaled < 0'))
hypothesis(fit_masteryincrease, c('Mastery_scaled:Agreeableness_scaled + EmotionAnger:Mastery_scaled:Agreeableness_scaled > 0'))
hypothesis(fit_masteryincrease, c('Mastery_scaled:Agreeableness_scaled + EmotionFocus:Mastery_scaled:Agreeableness_scaled > 0'))

# CONSCIENTIOUSNESS
hypothesis(fit_masteryincrease, c('Mastery_scaled:Conscientiousness_scaled < 0'))
hypothesis(fit_masteryincrease, c('Mastery_scaled:Conscientiousness_scaled + EmotionEnergy:Mastery_scaled:Conscientiousness_scaled < 0'))
hypothesis(fit_masteryincrease, c('Mastery_scaled:Conscientiousness_scaled + EmotionCalmness:Mastery_scaled:Conscientiousness_scaled < 0'))
hypothesis(fit_masteryincrease, c('Mastery_scaled:Conscientiousness_scaled + EmotionGloom:Mastery_scaled:Conscientiousness_scaled < 0'))
hypothesis(fit_masteryincrease, c('Mastery_scaled:Conscientiousness_scaled + EmotionGuilt:Mastery_scaled:Conscientiousness_scaled > 0'))
hypothesis(fit_masteryincrease, c('Mastery_scaled:Conscientiousness_scaled + EmotionSluggish:Mastery_scaled:Conscientiousness_scaled > 0'))
hypothesis(fit_masteryincrease, c('Mastery_scaled:Conscientiousness_scaled + EmotionAnxious:Mastery_scaled:Conscientiousness_scaled > 0'))
hypothesis(fit_masteryincrease, c('Mastery_scaled:Conscientiousness_scaled + EmotionDownhearted:Mastery_scaled:Conscientiousness_scaled > 0'))
hypothesis(fit_masteryincrease, c('Mastery_scaled:Conscientiousness_scaled + EmotionNervous:Mastery_scaled:Conscientiousness_scaled > 0'))
hypothesis(fit_masteryincrease, c('Mastery_scaled:Conscientiousness_scaled + EmotionAnger:Mastery_scaled:Conscientiousness_scaled > 0'))
hypothesis(fit_masteryincrease, c('Mastery_scaled:Conscientiousness_scaled + EmotionFocus:Mastery_scaled:Conscientiousness_scaled < 0'))

# EMOTIONAL STABILITY
hypothesis(fit_masteryincrease, c('Mastery_scaled:Stability_scaled < 0'))
hypothesis(fit_masteryincrease, c('Mastery_scaled:Stability_scaled + EmotionEnergy:Mastery_scaled:Stability_scaled < 0'))
hypothesis(fit_masteryincrease, c('Mastery_scaled:Stability_scaled + EmotionCalmness:Mastery_scaled:Stability_scaled > 0'))
hypothesis(fit_masteryincrease, c('Mastery_scaled:Stability_scaled + EmotionGloom:Mastery_scaled:Stability_scaled < 0'))
hypothesis(fit_masteryincrease, c('Mastery_scaled:Stability_scaled + EmotionGuilt:Mastery_scaled:Stability_scaled < 0'))
hypothesis(fit_masteryincrease, c('Mastery_scaled:Stability_scaled + EmotionSluggish:Mastery_scaled:Stability_scaled > 0'))
hypothesis(fit_masteryincrease, c('Mastery_scaled:Stability_scaled + EmotionAnxious:Mastery_scaled:Stability_scaled > 0'))
hypothesis(fit_masteryincrease, c('Mastery_scaled:Stability_scaled + EmotionDownhearted:Mastery_scaled:Stability_scaled < 0'))
hypothesis(fit_masteryincrease, c('Mastery_scaled:Stability_scaled + EmotionNervous:Mastery_scaled:Stability_scaled > 0'))
hypothesis(fit_masteryincrease, c('Mastery_scaled:Stability_scaled + EmotionAnger:Mastery_scaled:Stability_scaled < 0'))
hypothesis(fit_masteryincrease, c('Mastery_scaled:Stability_scaled + EmotionFocus:Mastery_scaled:Stability_scaled < 0'))
```

```
# EXTRAVERSION
hypothesis(fit_masteryincrease, c('Extraversion_scaled:Mastery_scaled < 0'))
hypothesis(fit_masteryincrease, c('Extraversion_scaled:Mastery_scaled + Extraversion_scaled:EmotionEnergy:Mastery_scaled < 0'))
hypothesis(fit_masteryincrease, c('Extraversion_scaled:Mastery_scaled + Extraversion_scaled:EmotionCalmness:Mastery_scaled < 0'))
hypothesis(fit_masteryincrease, c('Extraversion_scaled:Mastery_scaled + Extraversion_scaled:EmotionGloom:Mastery_scaled < 0'))
hypothesis(fit_masteryincrease, c('Extraversion_scaled:Mastery_scaled + Extraversion_scaled:EmotionGuilt:Mastery_scaled > 0'))
hypothesis(fit_masteryincrease, c('Extraversion_scaled:Mastery_scaled + Extraversion_scaled:EmotionSluggish:Mastery_scaled > 0'))
hypothesis(fit_masteryincrease, c('Extraversion_scaled:Mastery_scaled + Extraversion_scaled:EmotionAnxious:Mastery_scaled > 0'))
hypothesis(fit_masteryincrease, c('Extraversion_scaled:Mastery_scaled + Extraversion_scaled:EmotionDownhearted:Mastery_scaled < 0'))
hypothesis(fit_masteryincrease, c('Extraversion_scaled:Mastery_scaled + Extraversion_scaled:EmotionNervous:Mastery_scaled > 0'))
hypothesis(fit_masteryincrease, c('Extraversion_scaled:Mastery_scaled + Extraversion_scaled:EmotionAnger:Mastery_scaled > 0'))
hypothesis(fit_masteryincrease, c('Extraversion_scaled:Mastery_scaled + Extraversion_scaled:EmotionFocus:Mastery_scaled < 0'))

# OPENNESS
hypothesis(fit_masteryincrease, c('Mastery_scaled:Openness_scaled < 0'))
hypothesis(fit_masteryincrease, c('Mastery_scaled:Openness_scaled + EmotionEnergy:Mastery_scaled:Openness_scaled < 0'))
hypothesis(fit_masteryincrease, c('Mastery_scaled:Openness_scaled + EmotionCalmness:Mastery_scaled:Openness_scaled < 0'))
hypothesis(fit_masteryincrease, c('Mastery_scaled:Openness_scaled + EmotionGloom:Mastery_scaled:Openness_scaled < 0'))
hypothesis(fit_masteryincrease, c('Mastery_scaled:Openness_scaled + EmotionGuilt:Mastery_scaled:Openness_scaled < 0'))
hypothesis(fit_masteryincrease, c('Mastery_scaled:Openness_scaled + EmotionSluggish:Mastery_scaled:Openness_scaled < 0'))
hypothesis(fit_masteryincrease, c('Mastery_scaled:Openness_scaled + EmotionAnxious:Mastery_scaled:Openness_scaled > 0'))
hypothesis(fit_masteryincrease, c('Mastery_scaled:Openness_scaled + EmotionDownhearted:Mastery_scaled:Openness_scaled < 0'))
hypothesis(fit_masteryincrease, c('Mastery_scaled:Openness_scaled + EmotionNervous:Mastery_scaled:Openness_scaled > 0'))
hypothesis(fit_masteryincrease, c('Mastery_scaled:Openness_scaled + EmotionAnger:Mastery_scaled:Openness_scaled > 0'))
hypothesis(fit_masteryincrease, c('Mastery_scaled:Openness_scaled + EmotionFocus:Mastery_scaled:Openness_scaled < 0'))
```

```
# C.4 HYPOTHESIS TESTING FOR THE MASTERY DECREASE MODEL

# AGREEABLENESS
hypothesis(fit_masterydecrease, c('Mastery_scaled:Agreeableness_scaled > 0'))
hypothesis(fit_masterydecrease, c('Mastery_scaled:Agreeableness_scaled + EmotionEnergy:Mastery_scaled:Agreeableness_scaled > 0'))
hypothesis(fit_masterydecrease, c('Mastery_scaled:Agreeableness_scaled + EmotionCalmness:Mastery_scaled:Agreeableness_scaled < 0'))
hypothesis(fit_masterydecrease, c('Mastery_scaled:Agreeableness_scaled + EmotionGloom:Mastery_scaled:Agreeableness_scaled < 0'))
hypothesis(fit_masterydecrease, c('Mastery_scaled:Agreeableness_scaled + EmotionGuilt:Mastery_scaled:Agreeableness_scaled < 0'))
hypothesis(fit_masterydecrease, c('Mastery_scaled:Agreeableness_scaled + EmotionSluggish:Mastery_scaled:Agreeableness_scaled < 0'))
hypothesis(fit_masterydecrease, c('Mastery_scaled:Agreeableness_scaled + EmotionAnxious:Mastery_scaled:Agreeableness_scaled < 0'))
hypothesis(fit_masterydecrease, c('Mastery_scaled:Agreeableness_scaled + EmotionDownhearted:Mastery_scaled:Agreeableness_scaled < 0'))
hypothesis(fit_masterydecrease, c('Mastery_scaled:Agreeableness_scaled + EmotionNervous:Mastery_scaled:Agreeableness_scaled < 0'))
hypothesis(fit_masterydecrease, c('Mastery_scaled:Agreeableness_scaled + EmotionAnger:Mastery_scaled:Agreeableness_scaled < 0'))
hypothesis(fit_masterydecrease, c('Mastery_scaled:Agreeableness_scaled + EmotionFocus:Mastery_scaled:Agreeableness_scaled < 0'))

# CONSCIENTIOUSNESS
hypothesis(fit_masterydecrease, c('Mastery_scaled:Conscientiousness_scaled < 0'))
hypothesis(fit_masterydecrease, c('Mastery_scaled:Conscientiousness_scaled + EmotionEnergy:Mastery_scaled:Conscientiousness_scaled < 0'))
hypothesis(fit_masterydecrease, c('Mastery_scaled:Conscientiousness_scaled + EmotionCalmness:Mastery_scaled:Conscientiousness_scaled < 0'))
hypothesis(fit_masterydecrease, c('Mastery_scaled:Conscientiousness_scaled + EmotionGloom:Mastery_scaled:Conscientiousness_scaled > 0'))
hypothesis(fit_masterydecrease, c('Mastery_scaled:Conscientiousness_scaled + EmotionGuilt:Mastery_scaled:Conscientiousness_scaled > 0'))
hypothesis(fit_masterydecrease, c('Mastery_scaled:Conscientiousness_scaled + EmotionSluggish:Mastery_scaled:Conscientiousness_scaled > 0'))
hypothesis(fit_masterydecrease, c('Mastery_scaled:Conscientiousness_scaled + EmotionAnxious:Mastery_scaled:Conscientiousness_scaled > 0'))
hypothesis(fit_masterydecrease, c('Mastery_scaled:Conscientiousness_scaled + EmotionDownhearted:Mastery_scaled:Conscientiousness_scaled > 0'))
hypothesis(fit_masterydecrease, c('Mastery_scaled:Conscientiousness_scaled + EmotionNervous:Mastery_scaled:Conscientiousness_scaled > 0'))
hypothesis(fit_masterydecrease, c('Mastery_scaled:Conscientiousness_scaled + EmotionAnger:Mastery_scaled:Conscientiousness_scaled < 0'))
hypothesis(fit_masterydecrease, c('Mastery_scaled:Conscientiousness_scaled + EmotionFocus:Mastery_scaled:Conscientiousness_scaled < 0'))

# EMOTIONAL STABILITY
hypothesis(fit_masterydecrease, c('Mastery_scaled:Stability_scaled > 0'))
hypothesis(fit_masterydecrease, c('Mastery_scaled:Stability_scaled + EmotionEnergy:Mastery_scaled:Stability_scaled > 0'))
hypothesis(fit_masterydecrease, c('Mastery_scaled:Stability_scaled + EmotionCalmness:Mastery_scaled:Stability_scaled > 0'))
hypothesis(fit_masterydecrease, c('Mastery_scaled:Stability_scaled + EmotionGloom:Mastery_scaled:Stability_scaled > 0'))
hypothesis(fit_masterydecrease, c('Mastery_scaled:Stability_scaled + EmotionGuilt:Mastery_scaled:Stability_scaled > 0'))
hypothesis(fit_masterydecrease, c('Mastery_scaled:Stability_scaled + EmotionSluggish:Mastery_scaled:Stability_scaled > 0'))
hypothesis(fit_masterydecrease, c('Mastery_scaled:Stability_scaled + EmotionAnxious:Mastery_scaled:Stability_scaled > 0'))
hypothesis(fit_masterydecrease, c('Mastery_scaled:Stability_scaled + EmotionDownhearted:Mastery_scaled:Stability_scaled > 0'))
hypothesis(fit_masterydecrease, c('Mastery_scaled:Stability_scaled + EmotionNervous:Mastery_scaled:Stability_scaled > 0'))
hypothesis(fit_masterydecrease, c('Mastery_scaled:Stability_scaled + EmotionAnger:Mastery_scaled:Stability_scaled > 0'))
hypothesis(fit_masterydecrease, c('Mastery_scaled:Stability_scaled + EmotionFocus:Mastery_scaled:Stability_scaled > 0'))
```

```
#EXTRAVERSION
hypothesis(fit_masterydecrease, c('Extraversion_scaled:Mastery_scaled < 0'))
hypothesis(fit_masterydecrease, c('Extraversion_scaled:Mastery_scaled + Extraversion_scaled:EmotionEnergy:Mastery_scaled < 0'))
hypothesis(fit_masterydecrease, c('Extraversion_scaled:Mastery_scaled + Extraversion_scaled:EmotionCalmness:Mastery_scaled < 0'))
hypothesis(fit_masterydecrease, c('Extraversion_scaled:Mastery_scaled + Extraversion_scaled:EmotionGloom:Mastery_scaled < 0'))
hypothesis(fit_masterydecrease, c('Extraversion_scaled:Mastery_scaled + Extraversion_scaled:EmotionGuilt:Mastery_scaled < 0'))
hypothesis(fit_masterydecrease, c('Extraversion_scaled:Mastery_scaled + Extraversion_scaled:EmotionSluggish:Mastery_scaled < 0'))
hypothesis(fit_masterydecrease, c('Extraversion_scaled:Mastery_scaled + Extraversion_scaled:EmotionAnxious:Mastery_scaled < 0'))
hypothesis(fit_masterydecrease, c('Extraversion_scaled:Mastery_scaled + Extraversion_scaled:EmotionDownhearted:Mastery_scaled < 0'))
hypothesis(fit_masterydecrease, c('Extraversion_scaled:Mastery_scaled + Extraversion_scaled:EmotionNervous:Mastery_scaled < 0'))
hypothesis(fit_masterydecrease, c('Extraversion_scaled:Mastery_scaled + Extraversion_scaled:EmotionAnger:Mastery_scaled < 0'))
hypothesis(fit_masterydecrease, c('Extraversion_scaled:Mastery_scaled + Extraversion_scaled:EmotionFocus:Mastery_scaled < 0'))

# OPENNESS
hypothesis(fit_masterydecrease, c('Mastery_scaled:Openness_scaled > 0'))
hypothesis(fit_masterydecrease, c('Mastery_scaled:Openness_scaled + EmotionEnergy:Mastery_scaled:Openness_scaled < 0'))
hypothesis(fit_masterydecrease, c('Mastery_scaled:Openness_scaled + EmotionCalmness:Mastery_scaled:Openness_scaled > 0'))
hypothesis(fit_masterydecrease, c('Mastery_scaled:Openness_scaled + EmotionGloom:Mastery_scaled:Openness_scaled > 0'))
hypothesis(fit_masterydecrease, c('Mastery_scaled:Openness_scaled + EmotionGuilt:Mastery_scaled:Openness_scaled > 0'))
hypothesis(fit_masterydecrease, c('Mastery_scaled:Openness_scaled + EmotionSluggish:Mastery_scaled:Openness_scaled > 0'))
hypothesis(fit_masterydecrease, c('Mastery_scaled:Openness_scaled + EmotionAnxious:Mastery_scaled:Openness_scaled > 0'))
hypothesis(fit_masterydecrease, c('Mastery_scaled:Openness_scaled + EmotionDownhearted:Mastery_scaled:Openness_scaled > 0'))
hypothesis(fit_masterydecrease, c('Mastery_scaled:Openness_scaled + EmotionNervous:Mastery_scaled:Openness_scaled > 0'))
hypothesis(fit_masterydecrease, c('Mastery_scaled:Openness_scaled + EmotionAnger:Mastery_scaled:Openness_scaled < 0'))
hypothesis(fit_masterydecrease, c('Mastery_scaled:Openness_scaled + EmotionFocus:Mastery_scaled:Openness_scaled > 0'))
```

## References:


Cecchetti, G., Herff, S. A., and Rohrmeier, M. A. (2021). Musical syntactic structure improves memory for melody: evidence from the processing of ambiguous melodies. In Proceedings of the Annual Meeting of the Cognitive Science Society. Vol. 43 (No. CONF, pp. 2066-2071).

Dobrowohl, F. A., Milne, A. J., and Dean, R. T. (2019). Timbre preferences in the context of mixing music. Applied Sciences, 9(8), 1695.

Herff, S. A., Herff, C., Milne, A. J., Johnson, G. D., Shih, J. J., and Krusienski, D. J. (2020a). Prefrontal High Gamma in ECoG tags periodicity of musical rhythms in perception and imagination. Eneuro, 7(4).

Herff, S. A., Zhen, S., Yu, R., and Agres, K. R. (2020b). Age-dependent statistical learning trajectories reveal differences in information weighting. Psychology and Aging, 35(8), 1090.

Herff, S. A., Cecchetti, G., Taruffi, L., and Déguernel, K. (2021a). Music influences vividness and content of imagined journeys in a directed visual imagery task. Scientific reports, 11(1), 1-12.

Herff, S. A., Harasim, D., Cecchetti, G., Finkensiep, C., and Rohrmeier, M. A. (2021b). Hierarchical syntactic structure predicts listeners' sequence completion in music. In Proceedings of the Cognitive Science Conference. Vol 43 (No. CONF, pp. 903-909).

Jääskeläinen, T., López-Íñiguez, G., and Phillips, M. (2020). Music students' experienced workload, livelihoods and stress in higher education in Finland and the United Kingdom. Music Education Research, 22(5), 505-526.

Makowski, D., Ben-Shachar, M. S., Chen, S. H. A., and Lüdecke, D. (2019). Indices of Effect Existence and Significance in the Bayesian Framework. Frontiers in Psychology. 10:2767

Marsman, M., and Wagenmakers, E-J. (2017). Three insights from a Bayesian Interpretation of the One-Sided P Value. Educational and Psychological Measurement. Vol. 77(3), 529-539.

MacRitchie, J., Breaden, M., Milne, A. J., and McIntyre, S. (2020a). Cognitive, motor and social factors of music instrument training programs for older adults' improved wellbeing. Frontiers in psychology, 10, 2868.

Milne, A. J., and Herff, S. A. (2020). The perceptual relevance of balance, evenness, and entropy in musical rhythms. Cognition, 203, 104233

Smit, E. A., Dobrowohl, F. A., Schaal, N. K., Milne, A. J., and Herff, S. A. (2020). Perceived emotions of harmonic cadences. Music and Science, 3, 2059204320938635.

Smit, E. A., and Milne, A. J. (2021). The need for composite models of music perception: Consonance in tuning systems (familiar or unfamiliar) cannot be explained by a single predictor. Music Perception: An Interdisciplinary Journal, 38(3), 335-336.
